# The impact of opening dedicated clinics on disease transmission during an influenza pandemic

**DOI:** 10.1371/journal.pone.0236455

**Published:** 2020-08-06

**Authors:** Pengyi Shi, Jia Yan, Pinar Keskinocak, Andi L. Shane, Julie L. Swann

**Affiliations:** 1 Krannert School of Management, Purdue University, West Lafayette, Indiana, United States of America; 2 School of Industrial and Systems Engineering, Georgia Institute of Technology, Atlanta, Georgia, United States of America; 3 Division of Infectious Diseases, Department of Pediatrics, Emory University and Children’s Healthcare of Atlanta, Atlanta, Georgia, United States of America; 4 Department of Industrial and Systems Engineering, North Carolina State University, Raleigh, North Carolina, United States of America; University of Hong Kong, HONG KONG

## Abstract

Dedicated clinics can be established in an influenza pandemic to isolate people and potentially reduce opportunities for influenza transmission. However, their operation requires resources and their existence may attract the worried-well. In this study, we quantify the impact of opening dedicated influenza clinics during a pandemic based on an agent-based simulation model across a time-varying social network of households, workplaces, schools, community locations, and health facilities in the state of Georgia. We calculate performance measures, including peak prevalence and total attack rate, while accounting for clinic operations, including timing and location. We find that opening clinics can reduce disease spread and hospitalizations even when visited by the worried-well, open for limited weeks, or open in limited locations, and especially when the clinics are in operation during times of highest prevalence. Specifically, peak prevalence, total attack rate, and hospitalization reduced 0.07–0.32%, 0.40–1.51%, 0.02–0.09%, respectively, by operating clinics for the pandemic duration.

## Introduction

During the H1N1 influenza pandemic in 2009–2020, many people visited health facilities to seek diagnoses and treatment [1]. Visits to emergency departments (EDs) surge, which might result in opportunities for transmission to others. As a result, some facilities chose to dedicate space and resources to the establishment of clinics, which could diagnose and manage people with known or suspected influenza infections to divert them from EDs [2–4]. Dedicated influenza clinics could help to separate people with influenza-like illness (“ILI patients”) from other people seeking care for a non-ILI diagnosis (“non-ILI patients”), and thus reduce transmission to uninfected people who had the potential for a severe ILI manifestation if exposed. However, dedicated influenza clinics required human and material resources at a time when a system would be operating at full capacity. Additionally, dedicated influenza clinics could attract the worried-well, that is people who do not have the flu but are worried enough that they visit the flu clinic to be sure, seeking reassurance, utilizing resources, and potentially exposing themselves to others [5].

Two recent observational studies emphasized the importance of dedicated clinics during an influenza pandemic. FitzGerald et al. [6] reviewed the impact of the 2009 H1N1 influenza on ED operation in Australia. They concluded that dedicated influenza clinics could help manage people in an influenza pandemic and noted the importance of personal protective equipment and antivirals therapy in disease management. An observational retrospective study [7] in Taiwan found that a dedicated influenza clinic external to an ED could reduce the length of stay compared to regular ED services. However, both studies were observational, so it is difficult to quantify the impact of dedicated clinics during an influenza pandemic under different scenarios, project the resources needed, or compare dedicated clinics to other interventions.

In this study, we utilized an agent-based simulation to evaluate the impact of dedicated influenza clinics functioning for the duration of the pandemic versus for a limited time. We evaluated the changes in the prevalence of infection, the total attack rate in population at risk, hospitalizations, and transmission of infections in hospitals along with the resources needed to operate the clinics for different periods of time. Agent-based simulations have been widely used to model the spread of influenza in prior studies [8–13]; however, these models have not captured disease transmission occurring specifically in health facilities. A key feature of our study is that dedicated clinics may take time before they can be open, and they may not be open throughout the disease spread or across all locations. We accounted for people at higher risk of developing flu-related complications, e.g., young children, the elderly, pregnant women, and people with existing medical conditions [14], who seek healthcare at greater rates than lower-risk people. We also allowed for influenza clinics to bring together people who have influenza-like illnesses but may or may not have the flu, denoted at the “worried-well”. We compared the impact of dedicated influenza clinics with and without extensive use of masks in health facilities. We modeled disease spread in households, workplaces and schools, and the community among census tracts and counties in the state of Georgia [15] and quantified the impact of dedicated influenza clinics on transmission.

## Methods

Our agent-based simulation model included two critical components: (i) the disease progression within each agent (individual) and (ii) the contact network. Each agent in our model corresponded to an individual with certain social and geographical characteristics. The full details of the model (e.g., specifics on mixing, transmission, contact networks, etc.) are available in the S1 File.

### (i) Disease progression

The progression of flu within an individual is described using a Susceptible-Exposed-Infectious-Recovered (SEIR) model [16–18]. We described the progression of influenza with a refined SEIR model [19–22], which divided the infectious stage into more detailed sub-stages. Each agent was assumed to be in one of the following states: susceptible (*S*), exposed (*E*), infectious and presymptomatic (*I*_*P*_), infectious and symptomatic (*I*_*S*_), infectious and asymptomatic (*I*_*A*_), infectious and hospitalized (*I*_*H*_), recovered (*R*) or dead (*D*). All agents started in the susceptible state. The transition diagram is found in [22]. Agents are classified according to five age groups: 0–5, 6–11, 12–18, 19–64, and 65+ years. We assumed high-risk agents, e.g., people with co-morbidities that made them more vulnerable to severe outcomes from the flu, had a higher frequency of healthcare visits, and the probability of being hospitalized if influenza was contracted than low-risk ones. Hospitalizations are considered a severe outcome of influenza, which is typically associated with high mortality for the patients and a longer duration of being infectious to their contacts. The age- and risk-level specific transition probabilities and duration in each (sub-)stage are in Table 1. As the number of high-risk individuals may not be known, we established lower and upper bounds ([LB, UB]) for children [12%, 24%] and adults [8%, 24%] who were likely in this category. The details of the estimation are presented in the S1 File. We also assumed that anyone who recovers from influenza during the time horizon of the model is recovered with immunity and cannot infect others.

**Table 1:** Notations of constants used in our agent-based simulation model.

Parameter	Description	Values	References
*p*_*A*_	Probability of a presymptomatic person becoming asymptomatic	0.4 for adults aged 19–64, 0.25 for others	[22–25]
*p*_*H*_	Probability of a symptomatic person being hospitalized	0.18 for low-risk young children aged 0–5, 0.06 for low-risk people aged 6–64, 0.12 for adults aged above 65	[22, 25]
		0.36 for high-risk children aged 0–5, 0.12 for high-risk children aged 6–18, other high-risk age groups are the same as low-risk people	
*p*_*D*_	Probability of death for a hospitalized patient	0.344 for young children aged 0–5, and adults aged above 65, 0.172 for others	[22, 26]
Duration of *E+I*_*P*_	Duration of exposed and presymptomatic stages	Weibull distribution with mean 1.48 days (including an offset of 0.5 days) and standard deviation 0.47 days	[22, 27]
Duration of *I*_*P*_	Duration of presymptomatic stage	0.5 days	[22, 27]
Duration of *I*_*S*_	Duration of symptomatic stage	Exponential distribution with mean 2.73 days	[22]
Duration of *I*_*A*_	Duration of asymptomatic stage	Exponential distribution with mean 1.64 days	[22]
Duration of *I*_*H*_	Duration of hospitalization stage	Exponential distribution with mean 14 days	[22, 27]
Household size	Distribution of number of agents in one household	1-person household: 10.33%;	[15]
		2-person household: 23.55%;	
		3-person household: 20.45%;	
		4-person household: 23%;	
		5-person household: 12.79%;	
		6-person household: 5.91%;	
		7-person household: 3.97%.	
Classroom size	Distribution of number of students in one classroom	Age 0–5: uniform distribution (9,19); 6–11: uniform distribution (15,25); 12–18: uniform (25,35).	[22, 28]
Workplace size	Distribution of number of staff in one workplace	Poisson distribution with mean 20 (upper bound 1000)	[22, 25]
Community size	Number of agents in each census tract	Values from data source; maximum: 29341, minimum: 218	[15]
q_n_	Frequency of healthcare encounters for non-ILI^a^ patients	1 per year for low-risk non-ILI^a^ patients, 4 per year for high-risk non-ILI^a^ patients	[29]

^a^ ILI: influenza-like illness.

This model has been validated against previous pandemics, and versions of it have been published in several other papers (See S1 File).

### (ii) Contact network

Agents could contact each other within their social groups, including household (*H*), community (*C*), peer groups (*G*), hospital (*D*), and dedicated influenza clinic (*F*) if open. The hospital consists of an emergency department (ED) for short-term acute care and impatients who are admitted (or hospitalized) for care for at least one night. The peer group refers to schools or workplaces (based on the age group of each agent), and the community group is used to capture random contacts such as in churches or stores [19, 21]. The size distribution of social groups is in Table 1.

The model represented agents at the level of census tracts, with each assigned to a household size based on census data for the tract. During the daytime, agents are assigned to schools (including daycare, preschools), workplaces, or by themselves based on their age groups (≤18, 19–64, 65+ years old, respectively). All agents interact within their household at night and in communities (e.g., grocery stores) during both day and night. If a young patient (≤18 years old) is symptomatic, the person will withdraw from school; if an adult patient is symptomatic, the person will withdraw from work with a probability of 0.5.

We assume each agent is associated with the closest of 152 short-term acute care, critical access (e.g., providing healthcare for common conditions in rural areas), or children’s hospitals in Georgia, and each hospital could establish up to one dedicated influenza clinic to serve individuals associated with the hospital. We acknowledge that some patients may present to general practitioners. We are focused here on cases that need a great level of care, or on patients sent by general practitioners. Health facilities might are by agents in two categories: *ILI patients* who visited health facilities because they showed ILI symptoms, and *non-ILI patients* who sought care for diagnoses other than ILI. ILI-patients included those who were infected (flu patients) plus some worried-well individuals who thought they might be infected. We assumed worried-wells were present only when dedicated influenza clinics were open and that the number of the worried-well was proportional to the number of flu patients in the same clinic on that day. *P*_*ww*_ denotes the ratio of worried-well to other ILI patients; values are shown in Table 2. The timing of care seeking for patients who have influenza is random within the period where they are infectious and showing symptoms. Patients who have influenza are hospitalized according to the disease progression and can be admitted for overnight stays from clinics, EDs, or from the community.

**Table 2:** Notation and levels of parameters for baseline and sensitivity analysis.

Notation	Description	Levels of parameters
Clinic switch	Switch to settings with/out clinics	• NC (baseline): no clinic
		• CL: opening clinics based on *T*_*C*_, *X*_*C*,_ and *L*_*C*_
*MX*	Mixing mode at night	• FM (baseline): mixing with family at night
		• PT: mixing with other patients at night
P_hr_	Proportion of high-risk people	• UB (baseline): 22% for the children and 24% for adults
		• LB: 12% for the children and 8% for adults
		Estimated based on (*22–23*), details in the S1 File
*v*_*f*_	Probability to visit hospitals and clinics for flu patients	• Medium (baseline): 25% for low-risk flu patients and 50% for high-risk flu patients if clinic is not open, and 50% for low-risk flu patients and 100% for high-risk flu patients if clinic is open
		• Low: 10% for low-risk flu patients and 20% for high-risk flu patients if clinic is not open, and 20% for low-risk flu patients and 40% for high-risk flu patients if clinic is open
		• High: 37.5% for low-risk flu patients and 75% for high-risk flu patients if clinic is not open, and 75% for low-risk flu patients and 100% for high-risk flu patients if clinic is open
		Estimated from [30]
*q*_*f*_	Frequency to visit hospitals and clinics for flu patients	*v*_*f*_ /duration of *I*_*S*_ • Medium (baseline), low and high values based on *v*_*f*_
*T*_*c*_	Initiation of operation date of clinic *C*	• Week 1 (baseline)
		• Week 4
		• Week 5
		• Week 6
		• Week 7
		• Week 8
		• Week 9
		• Week 10
*X*_*c*_	Durations of clinic *C*	• 1 year (baseline)
		• 4 weeks
		• 8 weeks
*L*_*c*_	Location of clinic *C*	• Location Group 1—metropolitan Atlanta (Cherokee, Clayton, Cobb, DeKalb, Douglas, Fayette, Fulton, Gwinnett, Henry, Rockdale counties)
		• Location Group 2 –other locations
Mask types	Different masks have different effects on susceptibility and infectivity	• N95 (baseline): decreasing susceptibility by 20% and infectivity by 50%[31]
		• Surgical mask: decreasing susceptibility by 2% and infectivity by 5%[31]
*P*_*mk*_	Percentage of people wearing masks in hospitals and clinics	• No mask (baseline): 0%
		• 25%
		• 50%
		• 100%
Initial *R*_*0*_	Reproductive rate defined as average number of secondary cases generated by each infected patient before interventions are introduced	• 1.5 (baseline)
		• 1.8 [22, 25, 27, 32, 33]
*P*_*ww*_	Number of worried-well over number of flu patients in the same clinic	• 0.5 (baseline)
		• 0.2

The interactions (or lack thereof) between ILI and non-ILI patients is partly determined by the time of day and whether a dedicated influenza clinic was open. Each day patients visited health facilities based on whether they had ILI symptoms or not and their risk level (low or high, Table 1). During the daytime, non-ILI patients only visited hospitals (not dedicated influenza clinics). ILI patients visited dedicated influenza clinics if they were open; otherwise, ILI patients visited hospitals and mixed with non-ILI patients. During the night, we assumed all clinics were closed; thus, all flu patients visited hospitals (e.g., EDs) if they needed care during this time. People seeking care at hospitals or clinics did not interact with their usual peer groups during the time of the healthcare consultation. Agents not hospitalized might have contact with their community group during day and night.

ILI patients may be hospitalized. Non-ILI patients remained in the hospital using length of stay (LOS) distributions: we assumed 92% stayed in the hospital for 6 hours on average, and 8% of them stayed for five days on average [29]. For non-hospitalized ILI patients, we assumed they were in hospital EDs and clinics for 6 hours and 3 hours on average, respectively. We also added a small random perturbation (less than 1.2 hours) to the average LOS for each patient to model the uncertainty. After the LOS, patients left the health facilities and returned to their routine contact network. See Tables 1 and 2 for the notations and parameters for baseline cases and sensitivity analysis.

For hospitalized patients, we considered two mixing modes at night with: (1) (MX) *mixing with family*, i.e., patients in the hospital interacted with their family members at night but with no other patients; (2) *mixing with patients*, i.e., ILI and non-ILI patients interacted in the same hospital without any household members.

Similar to other studies [19–22], we used calibration to estimate unknown transmission parameters, including coefficient of transmission, relative hazards of an infected agent in different disease stages, and social groups. Details are provided in the S1 File.

#### Settings of scenarios

We set the no-clinic scenario as the baseline and compared it to scenarios with clinics. The scenarios around clinics, length of time, and start week reflect operational decisions that may be impacted by the lead time necessary to organize resources. In this way, we can capture the lead time that may be associated with setting up dedicated clinics, and we capture the resources by measuring the total days of operation across multiple scenarios. The scenarios showing the effect of masks can be considered as a comparison intervention or related to hospital policies. Several other scenarios are used for sensitivity analysis, such as around the percentage of high-risk patients or reproductive rate.

We considered the temporal and spatial features of partially opening dedicated influenza clinics: initiation of operation date (*T*_*c*_), durations (*X*_*c*_), and locations (L_c_). In our model, clinics were categorized by location: clinics in metropolitan Atlanta (Cherokee, Clayton, Cobb, DeKalb, Douglas, Fayette, Fulton, Gwinnett, Henry, Rockdale counties) [34] as *Location Group I*, and clinics in other locations as *Location Group II*. The initiation of operation date and durations of clinic openings were predetermined in each experiment. Clinics within the same location group shared the same schedule. We calculated the clinic resource days by the number of counties open (10, 149, 159, or 0) times the number of weeks open (0, 4, 8, or 52) times five days per week for each scenario. The schedules of clinics are in Table 2.

While focusing on clinics, we also compared the effects of extensive use of masks by anyone in the hospitals or clinics. Two face masks are considered: surgical masks and N95. Compared to surgical masks, the N95 decreased susceptibility and infectivity nine times more strongly [31]. We assumed a certain percentage (*P*_*mk*_) of people in health facilities wore masks. See Table 2 for details.

Comparing scenarios, we determined the effects of opening clinics year-round (SCEN 1,29,31,33 versus SCEN 2,30,32,34), different clinic initiation dates and durations by location (SCEN 3–22), using masks in health facilities (SCEN 23–26,35–36), different mixing modes at night (SCEN 1,2,31,32 versus SCEN 29,30,33,34), and the proportion of high-risk people (SCEN 1,2,2930 versus SCEN 31,32,33,34),. Detailed descriptions of the scenarios are in Table 3. To minimize the stochastic effects during the initial phase of the outbreak, we seeded the model with 30 initial random cases. There were 30 replications for each scenario, where each replication simulated 365 days from 30 first infected cases randomly distributed in the network on day one.

**Table 3:** Settings of scenarios.

Scenario	Description	Level of parameters	Purpose
SCEN 1	Baseline case has no clinic (NC)	NC.	Baseline no clinic scenario
SCEN 2	Clinic, length open 52 (L52) starting week 1 (S1)	C.L52.S1	Baseline clinic scenario
			To determine the impact of one-year clinics compared to the baseline no clinic scenario
SCEN 3	Clinics opened for length 4 weeks (L4) starting week 4 (S4)	C.L4.S4	To determine the impact of initiation date for 4-week clinics
SCEN 4	Clinics opened for length 4 weeks (L4) starting week 5 (S5)	C.L4.S5	
SCEN 5	Clinics opened for length 4 weeks (L4) starting week 6 (S6)	C.L4.S6	
SCEN 6	Clinics opened for length 4 weeks (L4) starting week 7 (S7)	C.L4.S7	
SCEN 7	Clinics opened for length 4 weeks (L4) starting week 8 (S8)	C.L4.S8	
SCEN 8	Clinics opened for length 4 weeks (L4) starting week 9 (S9)	C.L4.S9	
SCEN 9	Clinics opened for length 4 weeks (L4) starting week 10 (S10)	C.L4.S10	
SCEN 10	Clinics opened for length 8 weeks (L8) starting week 4 (S4)	C.L8.S4	To determine the impact of initiation date for 8-week clinics
SCEN 11	Clinics opened for length 8 weeks (L8) starting week 5 (S5)	C.L8.S5	
SCEN 12	Clinics opened for length 8 weeks (L8) starting week 6 (S6)	C.L8.S6	
SCEN 13	Clinics opened for length 8 weeks (L8) starting week 7 (S7)	C.L8.S7	
SCEN 14	Clinics opened for length 8 weeks (L8) starting week 8 (S8)	C.L8.S8	
SCEN 15	Clinics opened for length 8 weeks (L8) starting week 9 (S9)	C.L8.S9	
SCEN 16	Clinics opened for length 8 weeks (L8) starting week 10 (S10)	C.L8.S10	
SCEN 17	Clinics opened in Location Group I for length 52 weeks staring week 1 and in Location Group II for length 4 weeks starting week 7	C.I L52.II L4	To determine the impact of initiation date, duration and locations of clinics
SCEN 18	Clinics opened in Location Group I for length 52 weeks starting week 1 and in Location Group II for length 8 weeks starting week 7	C.I L52.II L8	
SCEN 19	Clinics opened in Location Group I for length 52 weeks starting week 1 and in Location Group II no clinics were opened	C.I L52.II L0	
SCEN 20	Clinics opened in Location Group I for length 4 weeks starting week 7 and in Location Group II no clinics were opened	C.I L4.II L0	
SCEN 21	Clinics opened in Location Group I for length 8 weeks starting week 7 and in Location Group II for length 4 weeks starting week 7	C.I L8.II L4	
SCEN 22	Clinics opened in Location Group I for length 8 weeks starting week 7 and in Location Group II no clinics were opened	C.I L8.II L0	
SCEN 23	No clinic (NC), N95 masks are worn 100% of time	NC.95M 100	To determine the effect of N95
SCEN 24	Clinic (C), N95 masks are worn 100% of time	C.95M 100	*P*_*mk*_: percentage of people wearing masks in hospitals and clinics
SCEN 25	No clinic (NC), surgical masks (SM) are worn 100% of time	NC.SM 100	To determine the effect of surgical mask
SCEN 26	Clinic (C), surgical masks (SM) are worn 100% of time	C.SM 100	
SCENARIOS FOR FURTHER SENSITIVITY ANALYSIS			
SCEN 27	No clinic (NC) with high reproductive rate (Rhigh) of 1.8.	NC.Rhigh	To determine the effect of *R*_*0*_
SCEN 28	Clinic (C) with high reproductive rate (Rhigh) of 1.8.	C.Rhigh	
SCEN 29	No clinic (NC) where patients mix with other patients at night (PT)	NC.PT	To determine the impact of mixing modes at night
SCEN 30	C clinic (C) where patients mix with other patients at night (PT)	C.PT	
			PT: mixing with other patients at night
SCEN 31	No clinic (NC), lower bound of proportion of high-risk people (LB)	NC.LB	To determine the impact of proportion of high-risk people
SCEN 32	Clinic (C), lower bound of proportion of high-risk people (LB)	C.LB	
			LB: lower bound of proportion of high-risk people
SCEN 33	No clinic (NC) where patients mix with other patients at night (PT), lower bound of proportion of high-risk people (LB)	NC.PT.LB	
SCEN 34	Clinic (NC) where patients mix with other patients at night (PT), lower bound of proportion of high-risk people (LB)	C.PT.LB	
SCEN 35	No clinic (NC), N95 masks are worn 25% of time	NC.95M 25	To determine the effect of partially wearing N95
SCEN 36	No clinic (NC), N95 masks are worn 50% of time	NC.95M 50	
SCEN 37	No clinic (NC), low frequency to visit hospitals and clinics for flu patients (Hlow)	NC.Hlow	To determine the effect of frequency to visit hospitals/clinics
SCEN 38	Clinic (C), low frequency to visit hospitals and clinics for flu patients (Hlow)	C.Hlow	
SCEN 39	No clinic (NC), high frequency to visit hospitals and clinics for flu patients (Hhigh)	NC.Hhigh	
SCEN 40	Clinic (C), low frequency to visit hospitals and clinics for flu patients (Hhigh)	C.Hhigh	
SCEN 41	No clinic (NC), low number of worried-well patients over number of flu patients in the same clinic (WWlow)	NC.WWlow	To determine the effect of number of worried-well
SCEN 42	Clinic (C), low number of worried-well patients over number of flu patients in the same clinic (WWlow)	C.WWlow	

#### Performance measures

We compared the following criteria across scenarios:

- Peak prevalence of infected individuals (i.e., symptomatic and asymptomatic) and peak day (i.e., the first day of peak prevalence);
- Total attack rate (i.e., the cumulative percentage of individuals who have been infected by the virus);
- Total hospitalizations (i.e., the percentage of individuals who have ever been admitted for inpatient care);
- Total hospitalizations of children (i.e., the percentage of children who have been admitted for inpatient care);
- Infections in hospitals and clinics (i.e., the cumulative number of people who incurred infections in hospitals and clinics, including patients and companions).

For peak day, we used the median from the 30 replications as its estimator. For all other measures, we took the mean across 30 replications as the estimator. In addition, in the scenarios of timing and location, we reported the relative benefit achieved by partially opened clinics to that of one-year clinics as the ratio of the difference in performance measures of the relevant scenario (SCEN X) and SCEN 1 compared to that of SCEN 1 and 2, i.e., (SCEN1-SCENX)/(SCEN1-SCEN2). We used hospitalizations (which represent a severe outcome associated with influenza) as a proxy for mortality also.

#### Sensitivity analysis

We conducted a one-way sensitivity analysis using several parameters, including the frequency symptomatic flu patients visit health facilities (*q*_*f*_), the basic reproductive rate (*R*_*0*_) of influenza, and the proportion of worried-well people (*P*_*ww*_) with parameter values as presented in Table 2. The scenarios for sensitivity analysis (SCEN 29–42) are in Table 3.

We conducted two-sample paired t-tests in R (package version 3.2.2) to compare performance measures of pairs of scenarios and reported two-tailed p-values.

## Results

### Opening clinics for one year versus no clinics (Table 4, Figs 1 and 2)

**Fig 1:**
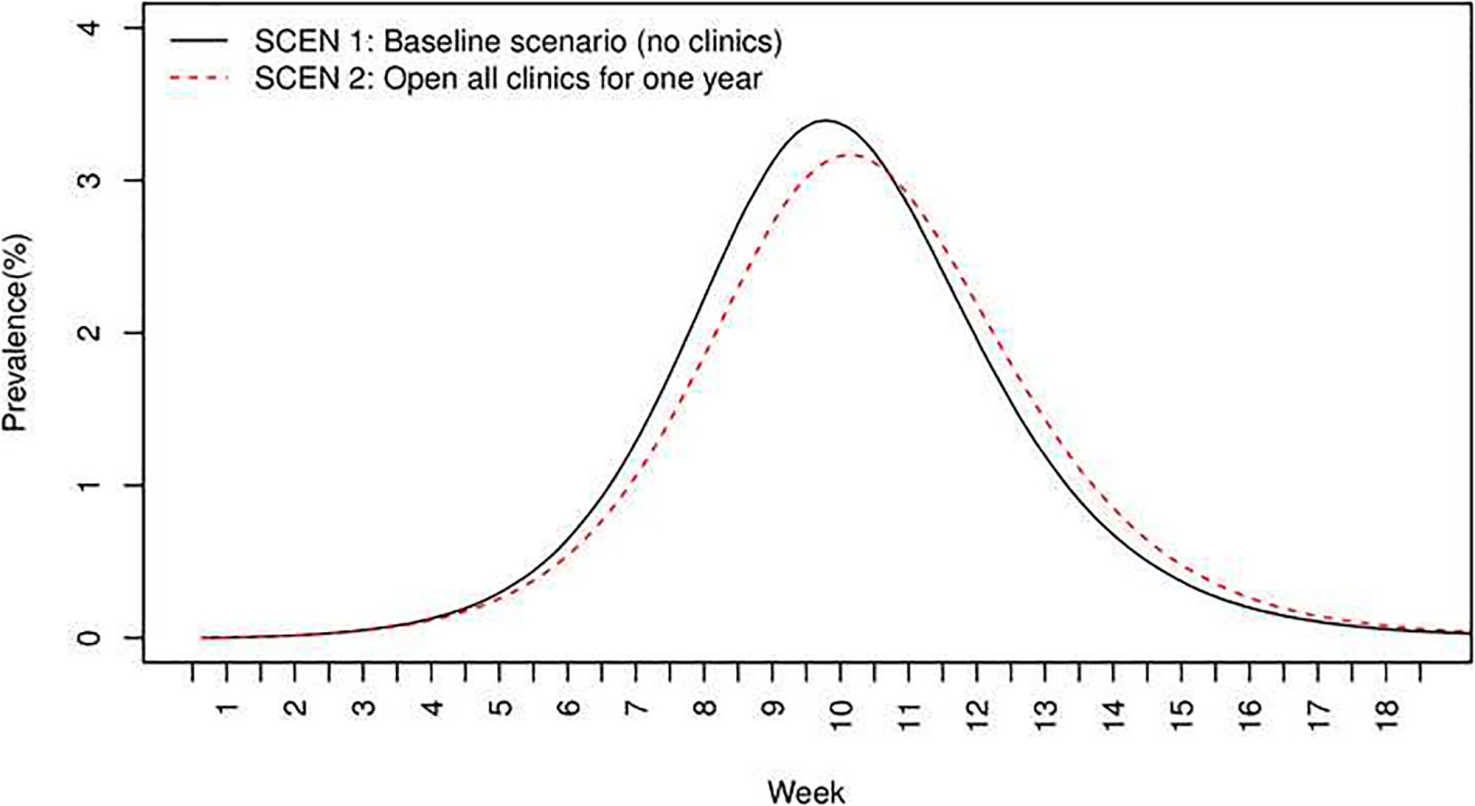
Prevalence (%) over time for SCEN 1 (no clinics) and SCEN 2 (one-year clinics).

**Fig 2:**
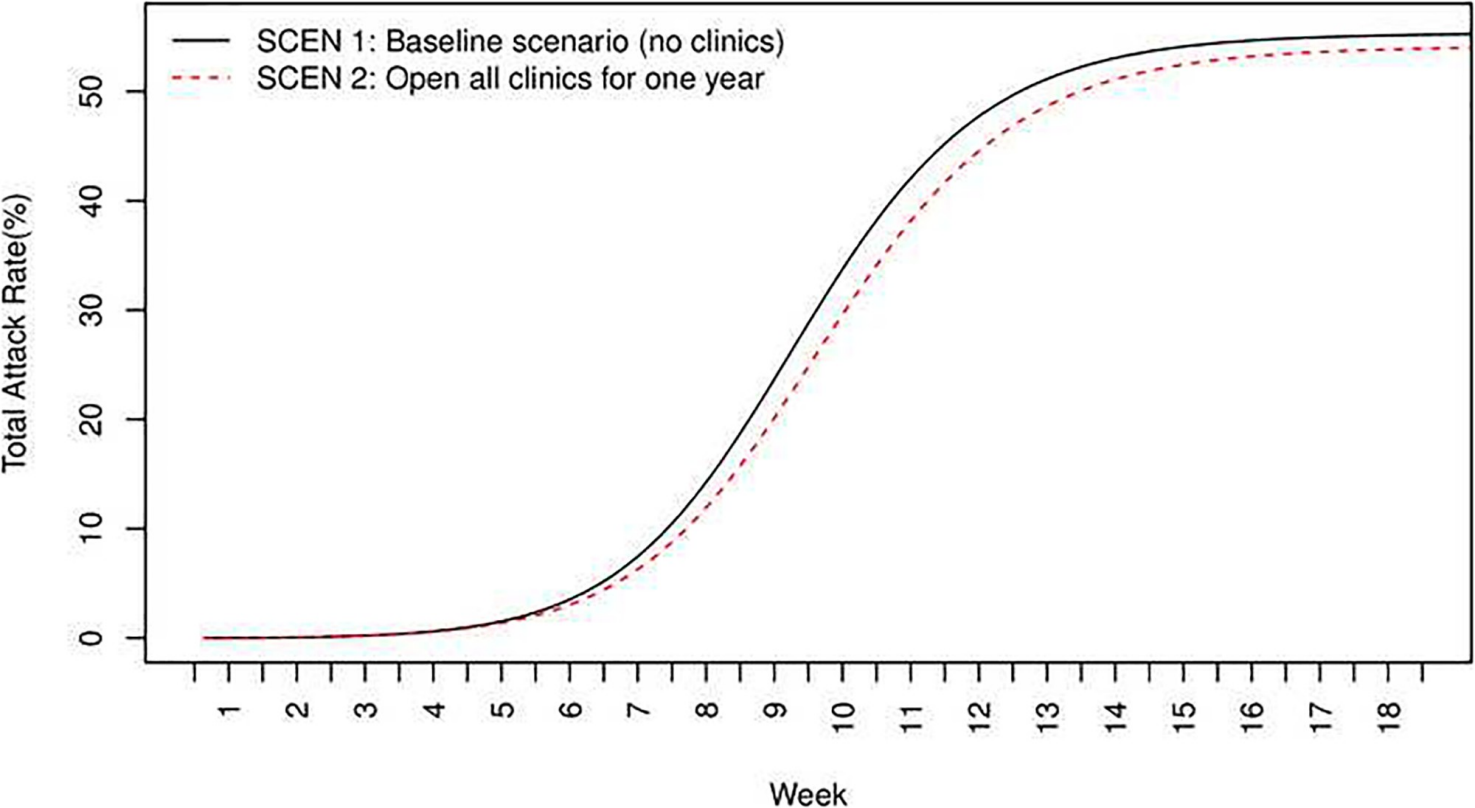
Total attack rates over time for SCEN 1 (no clinics) and SCEN 2 (one-year clinics).

**Table 4:** Performance measures for baseline scenario and scenarios with or without clinics, different mixing modes at night, and proportion of high-risk people.

Scenario	Level of Parameters	Peak prevalence (%)	Peak day	Total attack rate (%)	Hospitalization (%)	Hospitalization of children (%)	Infections in hospitals and clinics
SCEN 1	NC	3.41	65	55.82	3.07	5.21	330578
SCEN 2	C.L52.S1	3.19	68	54.64	3.01	5.08	281876

Compared with no clinics (SCEN 1), opening all clinics for the entire simulation period of transmission (SCEN 2) had lower peak prevalence of infected individuals (3.19% with clinics versus 3.41% without, *p*<0.001), lower total attack rate (54.64% versus 55.82%, *p*<0.001), lower hospitalizations (3.01% versus 3.07% for the entire population, 5.08% versus 5.21% for children, *p*<0.001 respectively), and fewer infections occurring in hospitals and clinics (281876 versus 330578, *p*<0.001). Opening clinics also tended to delay the peak day of prevalence (68 versus 65, *p*<0.001). Figs 1 and 2 present the prevalence and total attack rate, respectively, from week 1 to week 18. Note that the peak days were in week 10 (SCEN 1–2, Fig 1).

### Benefits of opening clinics with different timing and locations (Table 5, Figs 3 and 4)

**Fig 3:**
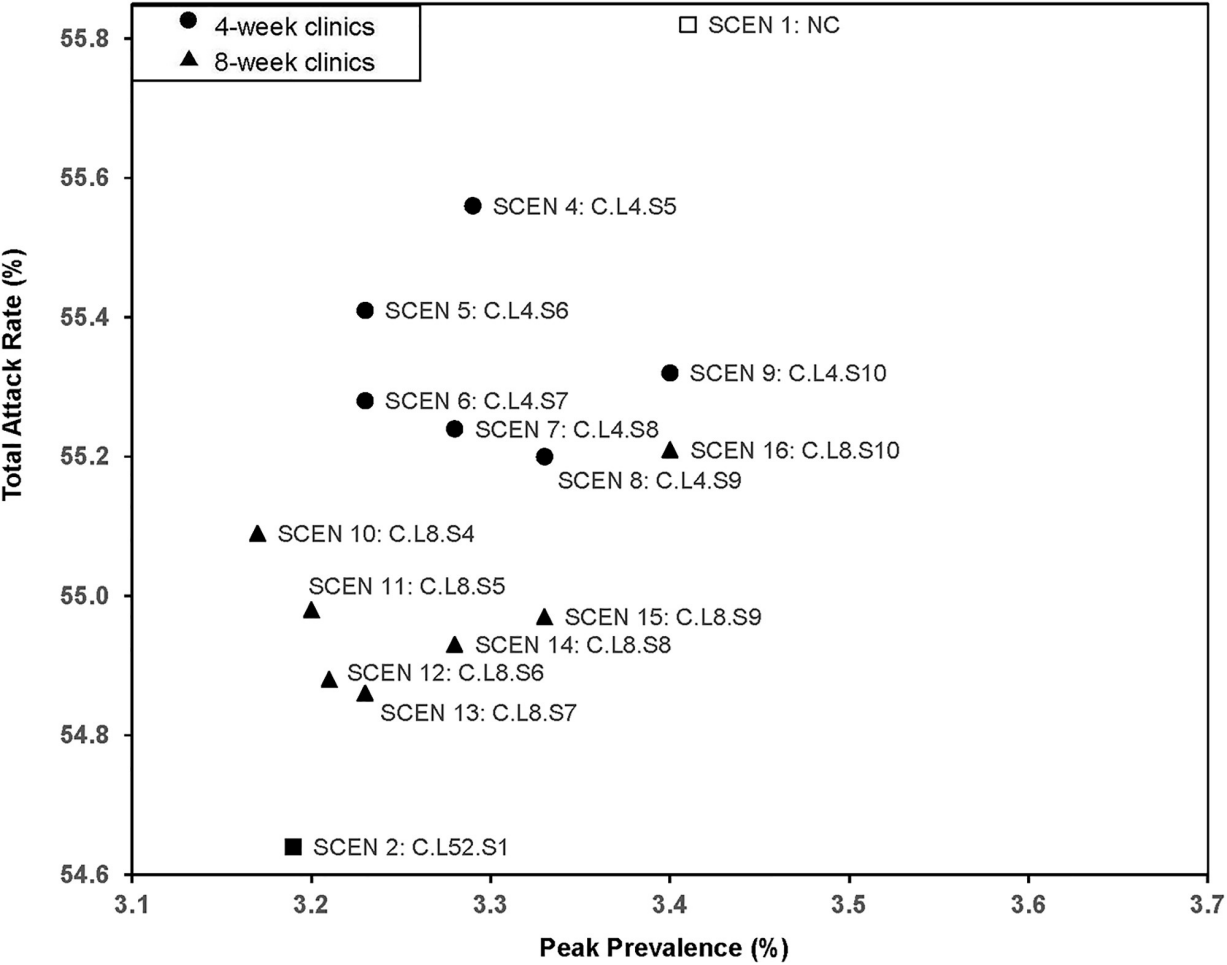
Total attack rate and peak prevalence of scenarios with 4(8)-week clinics open from weeks 4–10.

**Fig 4:**
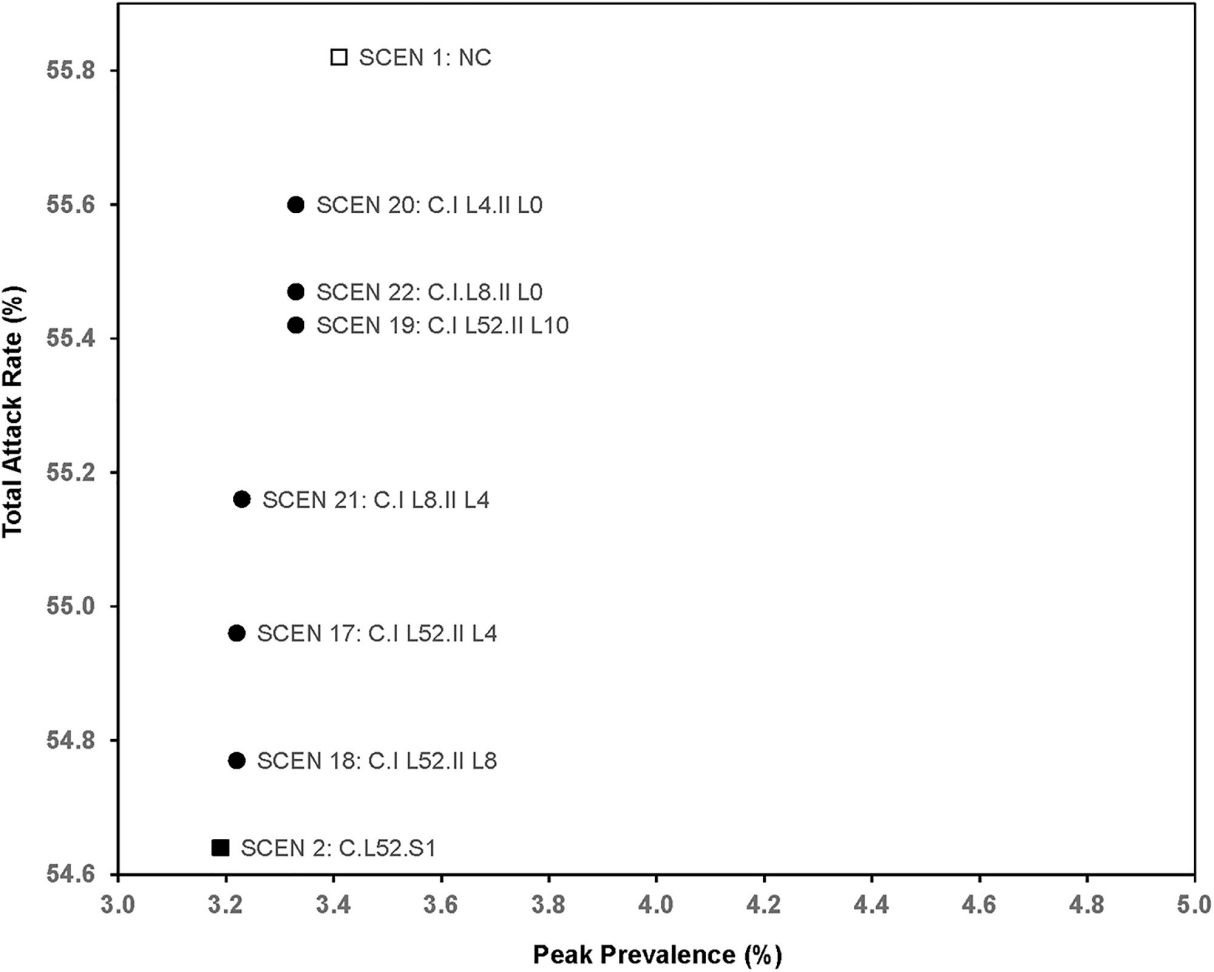
Total attack rate and peak prevalence of scenarios with location-based clinics.

**Table 5:** Performance measures for scenarios of clinics with different initiation of operation date, duration, or location along with baseline scenarios.

Scenario	Level of Parameters	Clinic Days	Peak prevalence (%)	Peak day	Total attack rate (%)	Hospitalization (%)	Hospitalization of children (%)	Infections in hospitals and clinics
SCEN 1	NC	0	3.41	65	55.82	3.07	5.21	330578
SCEN 2	C.L52.S1	41340	3.19	68	54.64	3.01	5.08	281876
SCEN 3	C.L4.S4	3180	3.31	67	55.63	3.08	5.20	319592
SCEN 4	C.L4.S5	3180	3.29	67	55.56	3.06	5.17	312562
SCEN 5	C.L4.S6	3180	3.23	67	55.41	3.05	5.17	308588
SCEN 6	C.L4.S7	3180	3.23	65	55.28	3.05	5.15	306487
SCEN 7	C.L4.S8	3180	3.28	65	55.24	3.05	5.15	308637
SCEN 8	C.L4.S9	3180	3.33	64	55.20	3.04	5.13	311290
SCEN 9	C.L4.S10	3180	3.40	64	55.32	3.05	5.16	315996
SCEN 10	C.L8.S4	6360	3.17	67	55.09	3.04	5.11	297205
SCEN 11	C.L8.S5	6360	3.20	66	54.98	3.03	5.12	293731
SCEN 12	C.L8.S6	6360	3.21	66	54.88	3.03	5.12	293154
SCEN 13	C.L8.S7	6360	3.23	65	54.86	3.02	5.10	294665
SCEN 14	C.L8.S8	6360	3.28	65	54.93	3.03	5.10	299343
SCEN 15	C.L8.S9	6360	3.33	64	54.97	3.03	5.12	305289
SCEN 16	C.L8.S10	6360	3.40	64	55.21	3.05	5.15	311689
SCEN 17	C.I L52.II L4	5580	3.22	66	54.96	3.02	5.11	296855
SCEN 18	C.I L52.II L8	8560	3.22	66	54.77	3.02	5.10	289756
SCEN 19	C.I L52.II L10	2600	3.33	66	55.42	3.06	5.17	311862
SCEN 20	C.I L4.II L0	200	3.33	65	55.60	3.06	5.16	319475
SCEN 21	C.I L8.II L4	3380	3.23	65	55.16	3.04	5.15	301911
SCEN 22	C.I.L8.II L0	400	3.33	65	55.47	3.06	5.17	315450

The detailed results of opening clinics with different timing and locations are listed in Table 5. For 4-week scenarios, the best results occurred in SCEN 5 (open at week 6) with the lowest peak prevalence of 3.23%, SCEN 6 (open at week 7) with lowest infections in hospitals and clinics (306487), and SCEN 8 (open at week 9), which had the lowest total attack rate of 55.20%. For the 8-week scenarios, the best results were in SCEN 10 (open at week 4) with the lowest peak prevalence of 3.17%, SCEN 12 (open at week 6) with lowest infections in hospitals and clinics and low total attack rates (54.88%), and SCEN 13 (open at week 7) with the lowest total attack rates (54.86%) and low infections incurred in hospitals and clinics (294665).

The initiation of operation date of dedicated influenza clinics had some impact on performance measures. Comparing results of SCEN 3–16 (Fig 3), starting at week 7 (SCEN 6 for 4-week and SCEN 13 for 8-week clinics) had a low total attack rate (55.28% and 54.86%, respectively), low hospitalizations (3.05% and 3.02%, respectively), and low infections incurred in hospitals and clinics (306487 and 294665, respectively). In comparison, starting earlier (SCEN 3, open weeks 4–7, compared to SCEN 6 open weeks 7–10) could be worse, unless clinics are open longer (SCEN 10, open weeks 4–11). Overall, scenarios covering periods when prevalence was increasing and at its peak tended to be best (SCEN 5–8 versus SCEN 3,4,9, and SCEN 10–15 versus SCEN 16).

Regarding locations and durations (Fig 4), the total attack rate for opening clinics in the metropolitan Atlanta region only for one year (SCEN 19) was 55.42%, which had a relative benefit of 34% of the reduction in total attack rate by opening clinics for one year in the entire state (SCEN 2). The results of SCEN 18, where clinics in the metropolitan Atlanta region were open for one year and in other locations for eight weeks starting at week 7, gained more of the relative benefits of opening clinics everywhere for a year, specifically 89%.

### Effects of masks compared to opening one-year clinics (Table 6, Fig 5)

**Fig 5:**
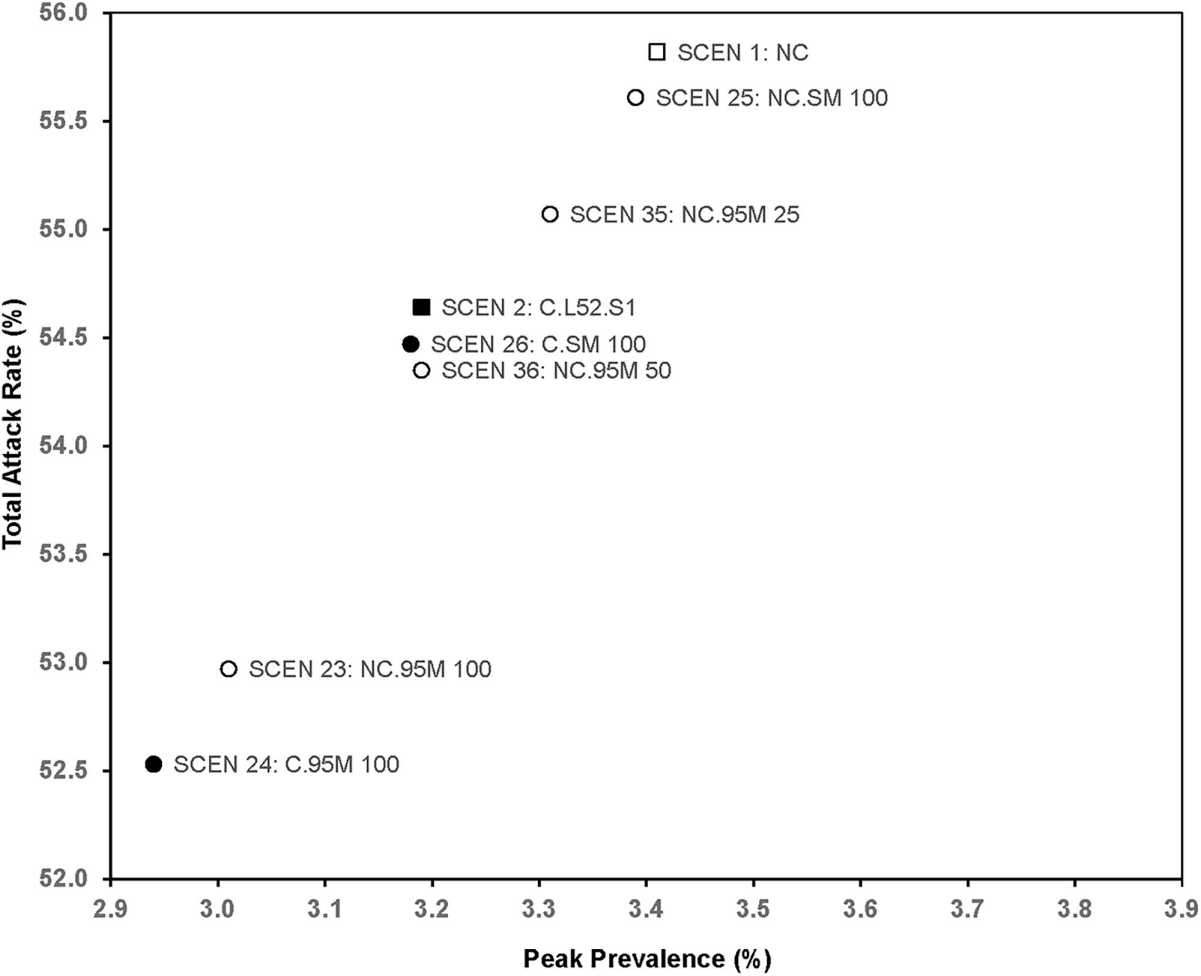
Total attack rate and peak prevalence of scenarios with masks.

**Table 6:** Performance measures for scenarios with masks.

Scenarios	Level of Parameters	Peak prevalence (%)	Peak day	Total attack rate (%)	Hospitalization (%)	Hospitalization of children (%)	Infections in hospitals and clinics
SCEN 23	NC.95M 100	3.01	68	52.97	2.90	4.90	169843
SCEN 24	C.95M 100	2.94	70	52.53	2.88	4.88	149232
SCEN 25	NC.SM 100	3.39	65	55.61	3.06	5.17	313313
SCEN 26	C.SM 100	3.18	68	54.47	3.00	5.07	268401
SCEN 35	NC.95M 25	3.31	66	55.07	3.04	5.14	287308
SCEN 36	NC.95M 50	3.19	67	54.35	2.99	5.05	246415

Opening one-year clinics (SCEN 2) had a stronger effect on total attack rate (54.64%) than fully wearing surgical masks (SCEN 25, *p*<0.001) and 25% wearing N95 (SCEN 35, *p*<0.001), while 50% wearing N95 (SCEN 36, *p*<0.001) was slightly better than fully opening clinics (SCEN 2). Wearing masks tended to reduce infections incurred in hospitals and clinics as well as hospitalizations. Fully wearing N95 (SCEN 23) dominated opening clinics for a full year (SCEN 2) in both peak prevalence and total attack rate (*p*<0.001), although the combined effect of masks and clinic could be greater (SCEN 24). Table 6 summarizes the effects of masks.

### Sensitivity analysis (Table 7, Figs 6–8)

**Fig 6:**
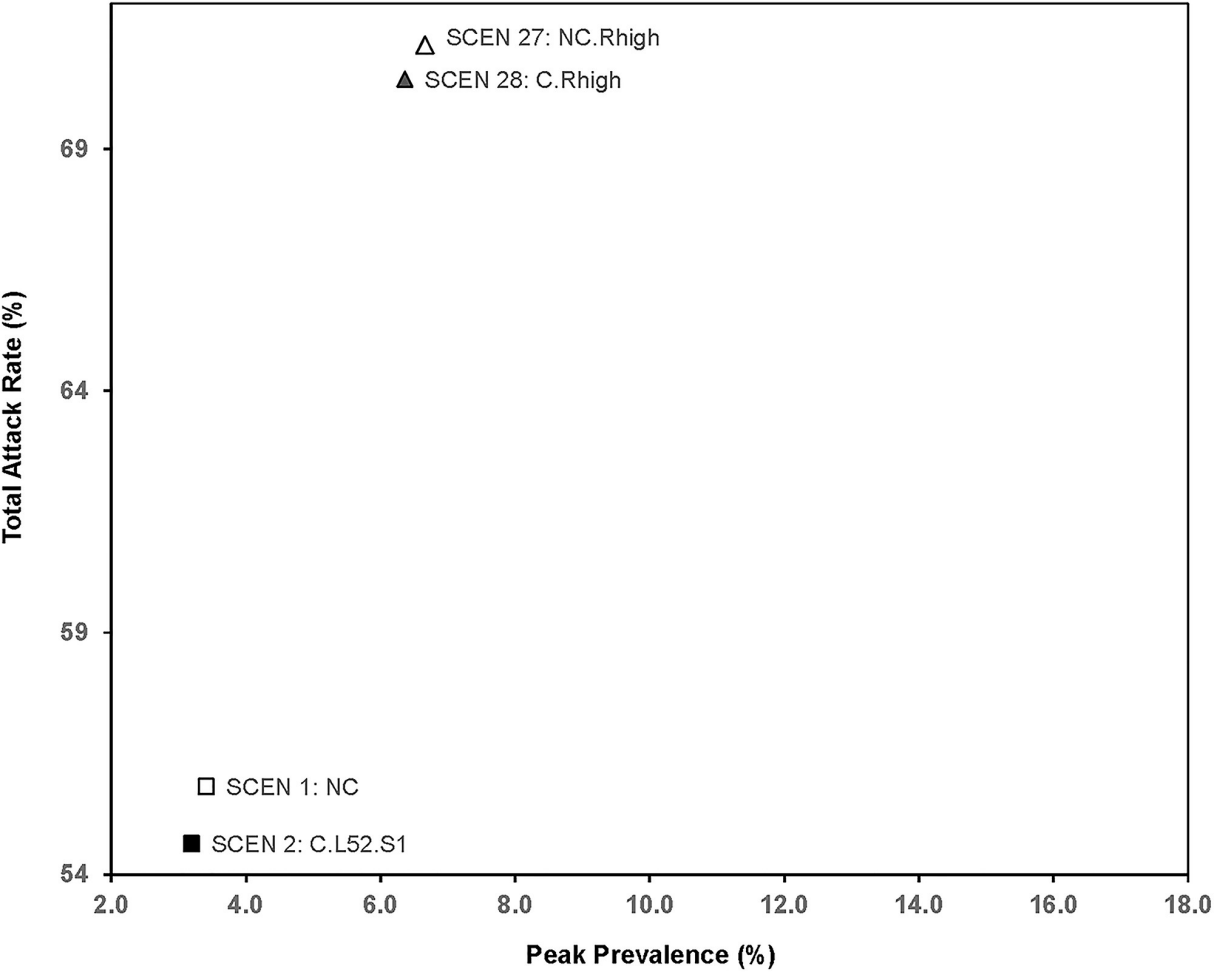
Total attack rate and peak prevalence of scenarios for sensitivity analysis (higher reproductive rate).

**Fig 7:**
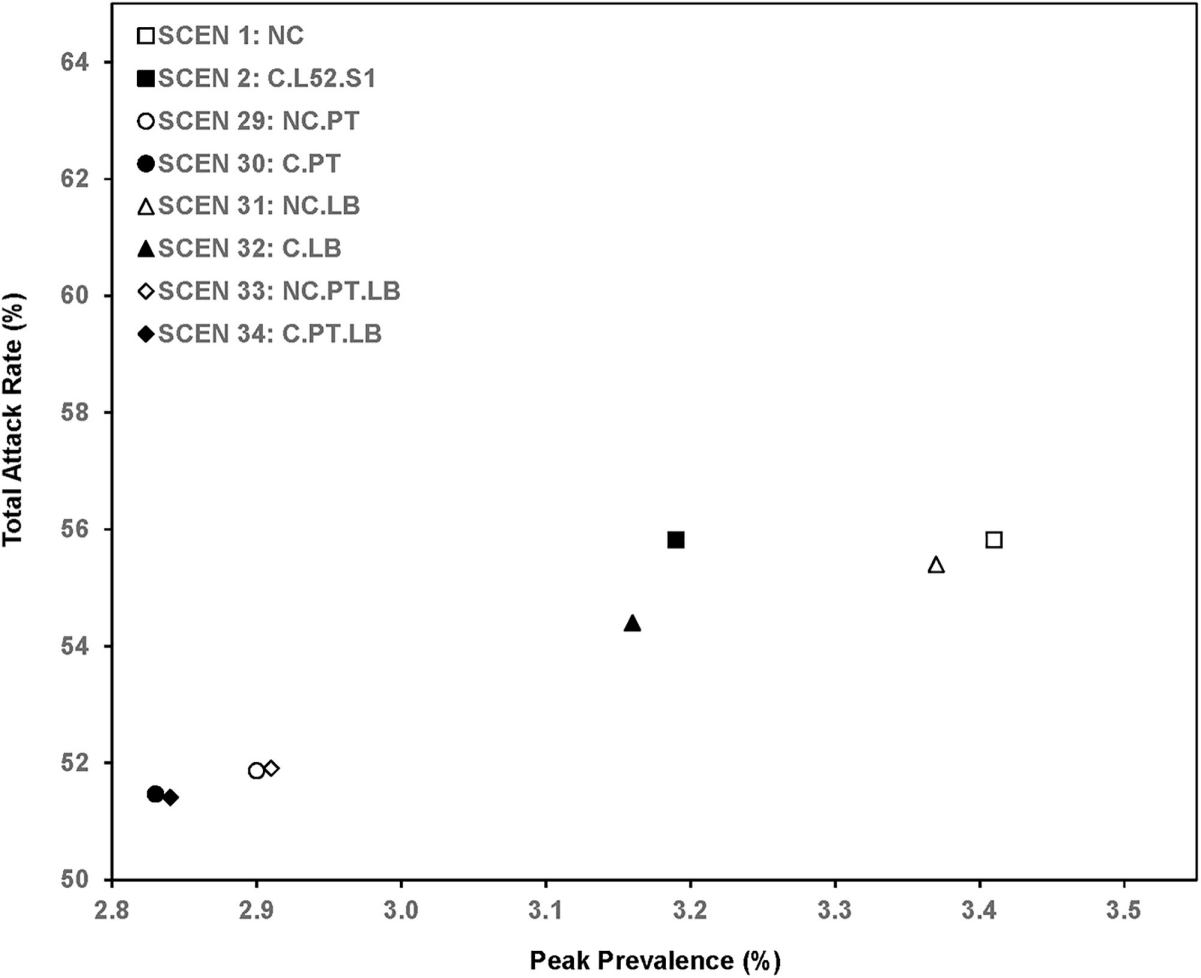
Total attack rate and peak prevalence of scenarios without clinics and with 1-year clinics.

**Fig 8:**
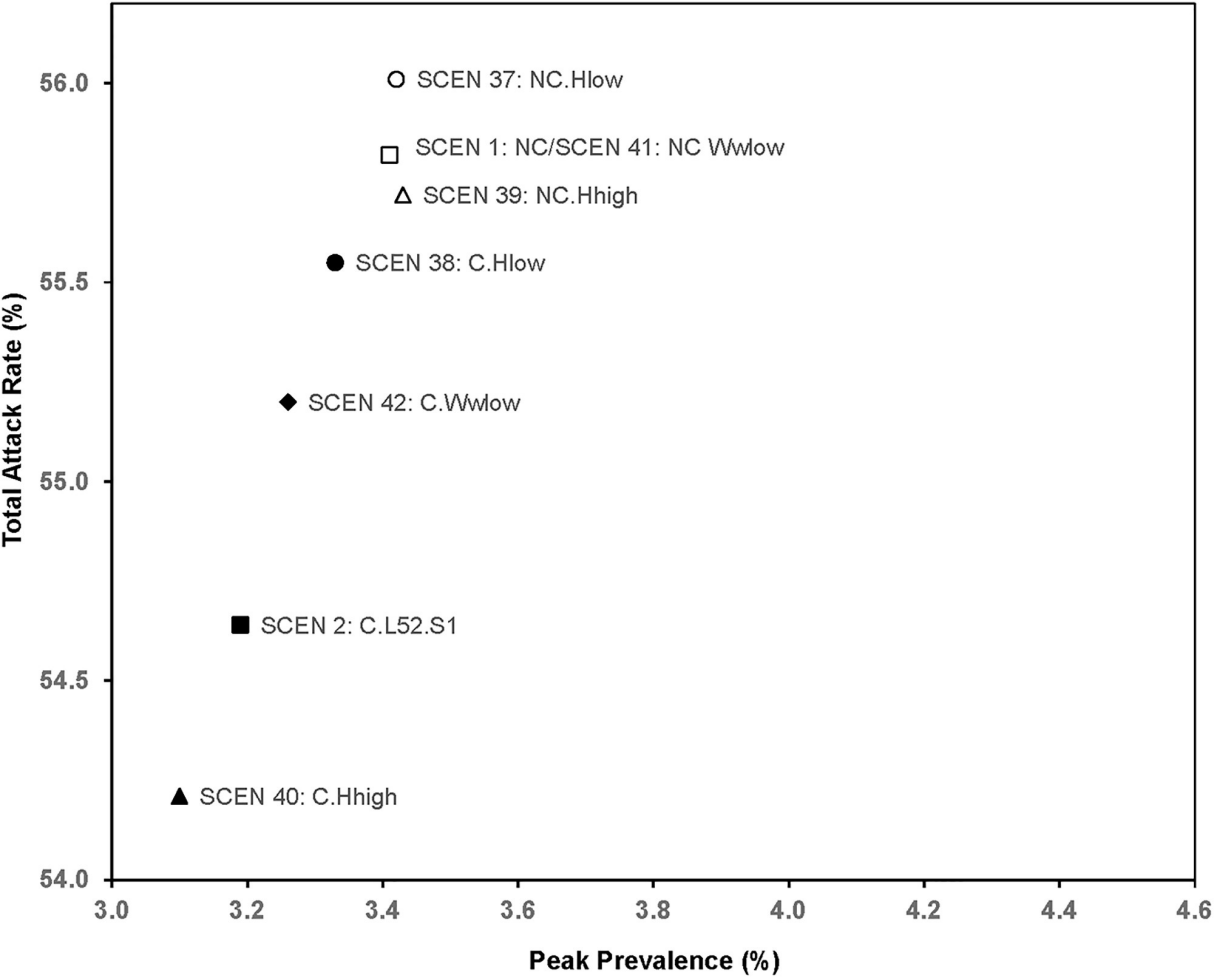
Total attack rate and peak prevalence of scenarios for low/high visit frequency, lower worried-well proportion.

**Table 7:** Sensitivity analysis.

Scenarios	Level of Parameters	Peak prevalence (%)	Peak day	Total attack rate (%)	Hospitalization (%)	Hospitalization of children (%)	Infections in hospitals and clinics
SCEN 27	NC.Rhigh	6.66	48	71.15	3.94	6.59	333422
SCEN 28	C.Rhigh	6.36	50	70.44	3.88	6.49	297954
SCEN 29	NC.PT	2.90	69	51.87	2.84	4.76	138049
SCEN 30	C.PT	2.83	71	51.47	2.82	4.76	104961
SCEN 31	NC.LB	3.37	66	55.40	2.93	4.74	292131
SCEN 32	C.LB	3.16	68	54.40	2.88	4.64	254583
SCEN 33	NC.PT.LB	2.91	70	51.91	2.73	4.39	118929
SCEN 34	C.PT.LB	2.84	70	51.41	2.71	4.37	93930
SCEN 37	NC.Hlow	3.42	66	56.01	3.09	5.21	309215
SCEN 38	C.Hlow	3.33	67	55.55	3.06	5.16	284940
SCEN 39	NC.Hhigh	3.43	65	55.72	3.07	5.19	342944
SCEN 40	C.Hhigh	3.10	69	54.21	2.98	5.04	281840
SCEN 41	NC.WWlow	3.41	65	55.82	3.07	5.21	330578
SCEN 42	C.WWlow	3.26	67	55.20	3.03	5.11	284555

For a higher reproduction rate (*R*_*0*_ = 1.8), the total attack rate increased from the base case (SCEN 1 with 55.82%) to 71.15% for no clinics (SCEN 27) or to 70.44% with 1-year clinics (SCEN 28). When patients mixed with family members at night, by opening clinics for one year, the total attack rate reduced 1.18% (SCEN 1 versus SCEN 2, *p*<0.001). When patients mixed with other patients at night, opening clinics reduced the total attack rate by 0.40% (SCEN 29 versus SCEN 30, *p*<0.001). Similarly for using the lower bound on high-risk patients, having clinics open for a year reduced the total attack rate by 1.00% when patients mixed with family members at night (SCEN 31 versus SCEN 32, *p*<0.001), and by 0.50% when patients mixed with other patients at night (SCEN 33 versus SCEN 34, *p*<0.001). The total attack rate and peak prevalence of the SCEN 1–2 and 29–34 are presented in Fig 7.

The total attack rates for low, medium, and high chance of visiting hospitals and clinics with 1-year clinics (SCEN 38, 2, and 40) were 55.55%, 54.64%, and 54.21%. If clinics brought in fewer worried-well (SCEN 42), the total attack rate was 55.20%, a little higher (*p*<0.001) than that for SCEN 2, which had more worried-well at *R*_*0*_ = 1.5. Table 7 summarizes the effects of clinics under various rates of visiting hospitals and clinics, a higher reproduction rate, or a lower proportion of worried-well.

Table 8 gives values for paired t-tests on multiple performance measures for many paired scenarios. We found statistically significant differences in the Total Attack Rate for all scenarios with a dedicated clinic as compared to a similar scenario without, at the 5% level or stronger. For a state that has a population of approximately 10 million, the difference in the baseline clinic case would be about 100,000 cases averted using 41,340 clinic days. For hospitalizations, we also found statistically significant differences for all scenarios with clinics except for SCEN 36, which was inconclusive. For a population of 10 million, the baseline case with clinics open would translate to about 6,000 hospitalizations averted. Comparing scenarios where clinics were open for a short time (4 weeks, SCEN 3) to a longer time (8 weeks, SCEN 10), the difference in total attack rate and hospitalizations would translate to about 50,000 cases averted and 4,000 hospitalizations averted, using 3,180 and 6,360 clinic days. The scenario with full clinics is better than that with clinics only in location set I (SCEN 19), translating into approximately 78,000 additional cases (or 5,000 hospitalizations) averted. Having full dedicated clinics was better than having 100% surgical masks (SCEN 25) with 97,000 cases and 5,000 hospitalizations averted, but having 100% N95 masks was better than having dedicated flu clinics, translating to approximately 167,000 cases and 11,000 hospitalizations averted.

**Table 8:** Paired t-tests, two-tailed (The alternative hypothesis is that the performance measures of Scenarios A and B are different; for measures other than peak day having a smaller performance measure is better.

Scenario A	Level of Parameters	Scenario B	Level of Parameters	Δ = Metric of A—Metric of B					
				(95% CI, p-value)					
				Δ Peak prevalence (%)	Δ Peak day	Δ Total attack rate (%)	Δ Hospitalization (%)	Δ Hospitalization of children (%)	Δ Infections in hospitals and clinics
	**No clinics vs. clinics**								
SCEN 1	NC	SCEN 2	C.L52.S1	0.23	-2.47	1.18	0.07	0.13	48702
				(0.20 to 0.26, < .001)	(-3.21 to -1.72, < .001)	(1.08 to 1.28, < .001)	(0.05 to 0.08, < .001)	(0.10 to 0.17, < .001)	(47287 to 50117, < .001)
SCEN 29	NC.PT	SCEN 30	C.PT	0.07	-1.60	0.40	0.02	0.01	33088
				(0.04 to 0.10, < .001)	(-2.41 to -0.79, < .001)	(0.28 to 0.53, < .001)	(0.01 to 0.03, 0.004)	(-0.02 to 0.04, NS)	(32449 to 33728, < .001)
SCEN 31	NC.LB	SCEN 32	C.LB	0.21	-2.33	1.00	0.05	0.10	37548
				(0.17 to 0.24, < .001)	(-3.16 to -1.51, < .001)	(0.90 to 1.10, < .001)	(0.03 to 0.07, < .001)	(0.07 to 0.13, < .001)	(35923 to 39174, < .001)
SCEN 33	NC.PT.LB	SCEN 34	C.PT.LB	0.07	-0.87	0.50	0.02	0.02	24999
				(0.04 to 0.11, < .001)	(-1.87 to 0.14, NS)	(0.37 to 0.63, < .001)	(0.01 to 0.04, < .001)	(-0.01 to 0.05, NS)	(24360 to 25639, < .001)
SCEN 37	NC.Hlow	SCEN 38	C.Hlow	0.09	-0.83	0.46	0.03	0.05	24276
				(0.05 to 0.13, < .001)	(-1.92 to 0.25, NS)	(0.36 to 0.56, < .001)	(0.02 to 0.05, < .001)	(0.01 to 0.08, 0.011)	(23111 to 25440, < .001)
SCEN 39	NC.Hhigh	SCEN 40	C.Hhigh	0.32	-3.90	1.51	0.09	0.15	61103
				(0.29 to 0.36, < .001)	(-4.69 to -3.11, < .001)	(1.40 to 1.61, < .001)	(0.07 to 0.11, < .001)	(0.11 to 0.20, < .001)	(59684 to 62523, < .001)
SCEN 27	NC.Rhigh	SCEN 28	C.Rhigh	0.30	-1.20	0.72	0.06	0.10	35468
				(0.24 to 0.36, < .001)	(-1.73 to -0.67, < .001)	(0.64 to 0.79, < .001)	(0.04 to 0.07, < .001)	(0.07 to 0.12, < .001)	(34077 to 36859, < .001)
SCEN 41	NC.WWlow	SCEN 42	C.WWlow	0.15	-1.50	0.62	0.04	0.10	46023
				(0.13 to 0.18, < .001)	(-2.20 to -0.80, < .001)	(0.50 to 0.74, < .001)	(0.03 to 0.06, < .001)	(0.07 to 0.12, < .001)	(44715 to 47331, < .001)
	**Open too early vs. later**								
SCEN 3	C.L4.S4	SCEN 6	C.L4.S7	0.08	1.50	0.35	0.03	0.05	13106
				(0.05 to 0.10, < .001)	(0.91 to 2.09, < .001)	(0.27 to 0.44, < .001)	(0.01 to 0.05, 0.001)	(0.01 to 0.10, 0.012)	(11579 to 14632, < .001)
	**Open for 4 weeks vs. 8 weeks**								
SCEN 3	C.L4.S4	SCEN 10	C.L8.S4	0.14	0.13	0.54	0.04	0.09	22388
				(0.12 to 0.16, < .001)	(-0.28 to 0.55, NS)	(0.43 to 0.65, < .001)	(0.03 to 0.06, < .001)	(0.05 to 0.13, < .001)	(21019 to 23756, < .001)
	**Location analysis**								
SCEN 19	C.I L52.II L0	SCEN 2	C.L52.S1	0.15	-1.77	0.78	0.05	0.10	29986
				(0.11 to 0.18, < .001)	(-2.64 to -0.89, < .001)	(0.67 to 0.89, < .001)	(0.04 to 0.07, < .001)	(0.07 to 0.13, < .001)	(28940 to 31032, < .001)
SCEN 18	C.1 L52.II L8	SCEN 2	C.L52.S1	0.03	-1.83	0.13	0.01	0.02	7880
				(0.00 to 0.06, NS)	(-2.77 to -0.89, < .001)	(0.04 to 0.21, 0.005)	(0.00 to 0.03, NS)	(-0.01 to 0.05, NS)	(6527 to 9234, < .001)
	**Mask analysis**								
SCEN 25	NC.SM 100	SCEN 2	C.L52.S1	0.21	-2.10	0.97	0.05	0.10	31437
				(0.17 to 0.24, < .001)	(-2.77 to -1.43, < .001)	(0.87 to 1.06, < .001)	(0.04 to 0.07, < .001)	(0.07 to 0.13, < .001)	(29995 to 32879, < .001)
SCEN 35	NC.94M 25	SCEN 2	C.L52.S1	0.13	-2.07	0.43	0.03	0.06	5432
				(0.10 to 0.16, < .001)	(-2.81 to -1.33, < .001)	(0.33 to 0.54, < .001)	(0.02 to 0.05, < .001)	(0.03 to 0.10, 0.001)	(4239 to 6625, < .001)
SCEN 2	C.L52.S1	SCEN 36	NC.95M 50	0.00	0.80	0.29	0.02	0.03	35460
				(-0.04 to 0.04, NS)	(-0.01 to 1.61, NS)	(0.17 to 0.40, < .001)	(0.00 to 0.04, NS)	(-0.01 to 0.07, NS)	(34041 to 36879, < .001)
SCEN 2	C.L52.S1	SCEN 23	NC.95M 100	0.18	-0.33	1.67	0.10	0.18	112033
				(0.14 to 0.22, < .001)	(-1.22 to 0.55, NS)	(1.56 to 1.78, < .001)	(0.09 to 0.12, < .001)	(0.15 to 0.21, < .001)	(110893 to 113172, < .001)
SCEN 23	NC.95M 100	SCEN 24	C.95M 100	0.07	-1.30	0.44	0.02	0.02	2061
				(0.03 to 0.11, 0.002)	(-2.22 to -0.38, 0.007)	(0.31 to 0.57, < .001)	(0.003 to 0.03, 0.020)	(-0.01 to 0.05, NS)	(1959 to 2163, < .001)
	**Sensitivity analysis**								
SCEN 28	C.Rhigh	SCEN 2	C.L52.S1	3.18	-17.87	15.79	0.88	1.41	16078
				(3.13 to 3.22, < .001)	(-18.61 to -17.12, < .001)	(15.69 to 15.90, < .001)	(0.86 to 0.89, < .001)	(1.38 to 1.45, < .001)	(14414 to 17743, < .001)
SCEN 27	NC.Rhigh	SCEN 1	NC	3.25	-16.60	15.33	0.86	1.38	2844
				(3.19 to 3.31, < .001)	(-17.16 to -16.04, < .001)	(15.26 to 15.40, < .001)	(0.85 to 0.88, < .001)	(1.35 to 1.41, < .001)	(1532 to 4156, < .001)
SCEN 42	C.WWlow	SCEN 2	C.L52.S1	0.07	-0.97	0.56	0.03	0.04	2679
				(0.04 to 0.11, < .001)	(-1.73 to -0.20, 0.015)	(0.46 to 0.66, < .001)	(0.01 to 0.04, 0.002)	(0.01 to 0.07, 0.019)	(1228 to 4130, < .001)

NS (not significant) denotes that the p-value>0.05).

## Discussion

The main goal of this research was to guide stakeholders on resource allocation by determining the impact of dedicated influenza clinics on the spread of disease during a pandemic under different scenarios. Dedicated clinics may also offer some additional benefits. For example, they could be used as a distribution point for medical countermeasures such as anti-virals. Additionally, such clinics could help with the availability of masks, which could be prioritized for personnel working in clinics dedicated to infectious disease. Clinics have the potential to draw the worried-well but also isolate flu patients from non-ILI patients. The results of comparing SCEN 1,29,31,33 (no clinic scenarios) versus 2, 30,32,33 (clinics open for one year) give an unequivocal conclusion that opening clinics for the duration of the epidemic can significantly reduce peak prevalence, total attack rate, hospitalizations, and the number of infections in healthcare facilities. The total attack rate is the lowest for scenarios that open clinics for the longest time period including SCEN 2, followed by SCEN 18, 12, and 13. The peak prevalence tends to be lowest for scenarios with clinics open the entire time period (SCEN 2). The peak can also be affected by clinics with specific opening times (e.g., SCEN 10), which is similar to putting an intervention in place for a limited time. The total attack rate is lowest when clinics are open a long time or cover the majority of the peak (e.g. SCEN 2 or SCEN 11–15).

While changing the attack rate from 55.82% to 51.41% may seem like a small change, for a population in one state the size of Georgia, this would translate to approximately 500,000 cases averted. Similarly, the change in hospitalization and mortality means that the best case intervention reduces hospitalization from 330,578 to 93,930 for a state the size of Georgia. The impact from dedicated clinics is not as much as a voluntary 8-week quarantine [see S1 File of reference 5] or vaccine that covered 20% or more of the population [35]. Yet averting more than 100,000 cases and thousands of hospitalizations may still be needed. In addition, having clinics open can delay the peak day of disease spread, which provides more time for the preparation of resources. These conclusions also hold for different visit rates to hospitals (SCEN 37,1,39 versus 38,2,40), fewer worried-well (SCEN 41 versus 42), and higher *R*_*0*_ (SCEN 27 versus 28).

In practice, with limited resources such as healthcare personnel, the operation of dedicated influenza clinics during a pandemic should maximize resource utilization. In the presence of dedicated clinics, there may be additional ways to serve the worried-well, such as encouraging them to call their practitioner for advice. Another advantage of the dedicated clinics is that they could free up resources for non-influenza related healthcare needs. We studied the effect of clinics opened after a period of time to examine the role that lead time has on outcomes. An alternative would be opening clinics after the epidemic passed some threshold of cases, where the threshold may occur sooner for high values of R_0_. This requires good knowledge of the R_0_ and the true cases in the population. Opening for one year may not be practical or needed. Rather, most of the effects of clinics can be achieved by carefully selecting start time and duration based on the pandemic dynamics. In particular, a goal should be to cover the periods when prevalence is increasing and at its peak. While in real-time, the peak is difficult to know, the usual rule of starting early enough (e.g., week 7) applies, and covering a number of weeks of high prevalence offers significant benefits even with limited resources (SCEN 13 vs. 2). Concentrating some clinical resources everywhere with additional resources in heavily populated areas is also a good strategy to consider (SCEN 18 vs. 2). If it is possible to reduce the lead time to set up clinics or reduce the resources associated with running a clinic, then additional benefits may be achieved, as demonstrated by the timing analysis.

Mixing patterns in hospitals and clinics impact disease control (Fig 7). If hospitalized flu-infected patients have contact with their uninfected family members at night (SCEN 1,2,31,32), transmission to household contacts and subsequently to members of peer groups of infected family members results in propagation of infection. Conversely, if flu-infected patients mix only with other flu-infected patients at night (SCEN 29,30,33,34), the hospitals can have a strong isolation effect, which can reduce disease spread in the population. Clinics may bring in worried-wells but may not necessarily increase infection transmission. The worried-well may affect disease spread in two ways. First, they are removed from their peer groups while they visit a healthcare facility and cannot get infected by their peer group. However, they have an elevated risk of being exposed to infections within clinics. When more worried-well enter clinics, the clinics create isolation for the worried-well away from their peer groups. Our model shows that increasing the number of worried-well (*P*_*ww*_ from 0.2 to 0.5, SCEN 42 versus SCEN 2) decreases the total attack rate (55.20% to 54.64%, *p*<0.001, Table 8, Fig 8), which implies that the reduction in infection by removal from peer groups is larger than the increased risk of infection by being present in a clinic. We find a reduction in hospitalizations, which is also a proxy for potential changes in mortality.

The results on masks are useful to consider. Having 50% of people in health facilities wearing N95 masks gives a similar reduction in attack rate as opening clinics for a full year (SCEN 36 and SCEN 2) while wearing 100% surgical masks (SCEN 25) is a little less effective than opening clinics fully (SCEN 2). Moreover, the resources required for masks would likely be much less than clinics, as long as a sufficient supply of N95 masks is stockpiled or available and if patients would wear masks according to the guidelines within healthcare facilities.

## Limitations

Modeling brings the usual limitations, including that it is based on assumptions such as mixing patterns and transmission rates. Our specific study also assumes people go to the regional hospital that is geographically close. We assume each hospital sets up one dedicated influenza clinic that has the resources to serve everyone. We validated our model against pandemics of previous decades but acknowledge that travel patterns and contact may have been different during that time. For some parameters, we do not have an accurate estimate, so we test different values to examine the robustness of our results. However, we could not test all combinations because of the large number of scenarios.

## Conclusions

Public health benefit results from the opening and operating of dedicated influenza clinics to diagnose and manage people with influenza-like illness during an influenza pandemic. Transmission of pandemic influenza infections is reduced when dedicated clinics are fully operational during the times of the greatest prevalence of the pandemic. Alternative strategies include operating clinics during the periods of highest prevalence during the pandemic, operating clinics based on population density, and wearing N95 in healthcare facilities.

## Supporting information

S1 File Appendix of the impact of opening dedicated clinics on disease transmission during an influenza pandemic. (PDF)
