# Supplementary material for: The impact of opening dedicated clinics on disease transmission during an influenza pandemic
Source: PLoS One. 2020 Aug 6;15(8):e0236455. doi: 10.1371/journal.pone.0236455 (PMC7410326; doi:10.1371/journal.pone.0236455)
Supplement: S1 File — (PDF) [file pone.0236455.s001.pdf]

# S1. Appendix of the Impact of Opening Dedicated Clinics on Disease Transmission during an Influenza Pandemic

## 1 Lower and Upper Bound (LB and UB) Estimations for High-Risk Children and Adults

As all asthma patients are considered high-risk [1] , we use a LB of 12% for children and LB of 8% for adults [2]. The UB is determined from our empirical data and arithmetic calculation. We find that 22% of the children are at high risks among those visiting the Children's Healthcare of Atlanta (the largest provider of pediatric services in Georgia) in year 2009 and set it as the UB for children. We estimate 24% as the UB of adults who are high-risk (denoted as $P_{hr,ad}$) based on (i) the high-risk proportion (denoted as $P_{hr,overall}$, 20.7%=62/299 from a CDC report[3]) in the entire population, (ii) the proportions of adults and children in the population (denoted as $P_{ad}, P_{ch}$ respectively), and (iii) the LB of high-risk children (denoted as $P_{hr,ch}$). Specifically, the following is the formula we use to derive the UB of adults:

$$P_{hr,ad} = (P_{hr,overall} - P_{hr,ch} \times P_{ch})/P_{ad} \leq (P_{hr,overall} - (\text{LB of } P_{hr,ch}) \times P_{ch})/P_{ad}.$$

## 2 Natural Disease Progression Model and Parameters

We use a detailed SEIR (Susceptible, Exposed, Infectious, and Recovered) model [4, 5, 6] to depict the disease progression of individuals based on their ages and risk groups. In particular, individuals are divided into five age groups: 0–5, 6–11, 12–18, 19–64, 65+. They are either low-risk or high-risk. Each individual can stay in one of the disease stages, i.e. susceptible ($S$), exposed but not infectious ($E$), presymptomatic ($I_P$), asymptomatic ($I_A$), symptomatic ($I_S$), hospitalized ($I_H$), recovered ($R$) and dead ($D$), at a certain time. The disease progression model is shown in Figure 1 of Ekici, Keskinocak and Swann[5]. All individuals start from susceptible stage. If infected, individuals become exposed but not infectious and then presymptomatic. Presymptomatic patients may not show any symptom with probability $p_A$ or develop symptoms with probability $1 - p_A$. Asymptomatic patients recovers for sure. Symptomatic patients may be hospitalized with probability $p_H$ or directly get recovered with probability $1 - p_H$. Hospitalized patients may die with probability $p_D$ or recover with probability $1 - p_D$. Patients are infectious when they are in presymptomatic, asymptomatic, symptomatic and hospitalized stages. Individuals who get recovered are immune to the disease. The age-and-risk-specific parameters are presented in Table 1.

Table 1. Natural disease progression parameters.

| Notations | Descriptions | Values | References |
|---|---|---|---|
| $p_A$ | Probability of infected individual to become asymptomatic | 0.4 for working adults (age 19–24) and 0.25 for others | [4, 7, 8, 9] |
| $p_H$ | Probability of symptomatic individual to be hospitalized | Among low-risk people, 0.18 for young children (age 0–5), 0.12 for elderly (age 65+), and 0.06 for others; among high-risk people, 0.36 for young children (age 0–5), 0.12 for children (age 5–18) and elderly (age 65+), and 0.06 for others. | [4, 9] |
| $p_D$ | Probability of hospitalized individual who becomes dead | 0.344 for children (age 0–5) and elderly (age 65+), 0.172 for for others | [4, 10] |
| Duration of $E + I_P$ | Length of exposed and presymptomatic stage | Weibull with mean 1.48 days and standard deviation 0.47 days and offset of 0.5 days | [4, 11] |
| Duration of $I_S$ | Length of symptomatic stage | Exponential with mean 2.7313 days | [4] |
| Duration of $I_A$ | Length of asymptomatic stage | Exponential with mean 1.63878 days | [4] |
| Duration of $I_H$ | Length of hospitalized stage | Exponential with mean 14 days | [4, 11] |
| Initial $R_0$ | Reproductive rate (average number of secondary cases generated by an infectious individual) before hospitals and clinics are introduced | 1.5, 1.8 | [4, 12, 9, 11, 13] |
| $\theta$ | Proportion of transmission that occurs at presymptomatic or asymptomatic stage | 0.3 | [4] |
| $\omega$ | Proportion of infections generated by individuals who are asymptomatic | 0.15 | [4] |
| $\gamma$ | Proportion of transmission that occurs outside the households | 0.7 | [12] |
| $\delta$ | Proportion of transmission outside the households that occurs in the community | 0.5 | [12] |

# 3 Contact Network and Parameters

In our simulation model, individuals contact others in social groups, including household ($H$), community ($C$), peer groups ($G$), hospitals ($D$), and flu clinics ($F$). Peer groups are classrooms for children and workplaces for adults. The average peer group sizes are 14, 20 and 30 for children in age groups 0–5, 6–11 and 12–18 respectively [14]. The workplace sizes for adults in age group 19–64 follow a truncated Poisson distribution with mean 20 and maximum 1000 [8]. Elderly with age 65 or above stay alone in his/her peer group. Initially, all individuals are susceptible. We randomly pick 30 individuals in the population to get infected.

Our simulation model determines the time of next infection and chooses a person to get infected based on the method of instantaneous FOI (Force Of Infection) in prior studies [4, 5, 6]. A higher instantaneous FOI experienced by a susceptible individual implies a higher probability the individual become infected in the social group. The instantaneous FOI experienced by the $i$th person in the day ($\lambda_i^D$) and in the night ($\lambda_i^N$) are calculated using the following formulas. Notations and calculations will be explained in

details following the formulas.

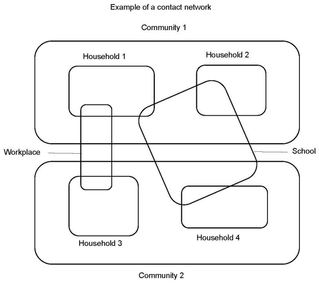


$$\lambda_i^D = \begin{cases} S_i \sum_{j=1}^N \delta_{ij}^D \epsilon_j^D m_j \bar{h}_{X_j} \bar{h}_D \bar{\beta}/n_i^D & \text{if } i \text{ is in the hospital mixing with family;} \\ S_i \sum_{j=1}^N \delta_{ij}^D \epsilon_j^D m_j \tilde{h}_{X_j} \tilde{\beta}/n_i^D & \text{if } i \text{ is in the hospital mixing with patients;} \\ S_i \sum_{j=1}^N \delta_{ij}^F \epsilon_j^F m_j \tilde{h}_{X_j} \tilde{\beta}/n_i^F & \text{if } i \text{ is in the clinic;} \\ S_i \sum_{j=1}^N (\delta_{ij}^G \epsilon_j^G m_j h_{X_j} h_G \beta & \\ \quad +\delta_{ij}^C m_j h_{X_j} h_C \beta / N_i^C) & \text{otherwise.} \end{cases}$$

$$\lambda_i^N = \begin{cases} S_i \sum_{j=1}^N \delta_{ij}^H m_j h_{X_j} \beta/n_i^{HA} & \text{if } i \text{ is in the hospital mixing with family;} \\ S_i \sum_{j=1}^N \delta_{ij}^D \epsilon_j^D m_j \tilde{h}_{X_j} \tilde{\beta}/n_i^D & \text{if } i \text{ is in the hospital mixing with patients;} \\ S_i \sum_{j=1}^N (\delta_{ij}^H m_j h_{X_j} \beta/n_i^{HA} & \\ \quad +\delta_{ij}^C m_j h_{X_j} h_C \beta / N_i^C) & \text{otherwise.} \end{cases}$$

In particular, $S_i$ is the susceptibility of the $i$th individual and $m_i$ is the infectivity of a symptomatic individual $i$. They are defined the same as prior papers [5, 6, 10] , i.e.,

$$S_i = \begin{cases} 0 & \text{if } i \text{ is not susceptible;} \\ 1.1036 & \text{if } i \text{ is a susceptible child;} \\ 0.9597 & \text{if } i \text{ is a susceptible adult.} \end{cases}$$

and

$$m_i = \begin{cases} 1.3158 & \text{if } i \text{ is a child;} \\ 0.8772 & \text{if } i \text{ is a adult.} \end{cases}$$

Moreover, $n_i^D$ and $n_i^F$ denote the number of individuals in the same hospital or clinic as the $i$th individual respectively. $n_i^{HA}$ is the number of active household members of the $i$th individual, which excludes those dead and hospitalized individuals in $i$th individual's household. $N_i^C$ is the number of individuals in the $i$th individual's community. $\delta_{ij}^H$, $\delta_{ij}^G$, $\delta_{ij}^C$, $\delta_{ij}^D$ and $\delta_{ij}^F$ are indicators: if $i$th and $j$th individuals are within the same social group (household, peer group, community, hospital, and clinic respectively), the variable is set to be 1; otherwise the variable is 0. We assume that all symptomatic children withdraw from their peer groups and symptomatic adults withdraw from work with probability 0.5. $\epsilon_j^G$, $\epsilon_j^D$ and $\epsilon_j^F$ are indicators of $j$th individual being in his/her peer group, hospital and clinic respectively.

In our model, we consider three major maxing patterns: (i) The basic-mixing routine, where individuals mix in their peer groups during daytime, in their household groups during the night, and have some random contacts in their community groups, e.g. church and grocery stores, both day and night; (ii) The hospital/clinic-mixing routine and mixing with family mode at night, where individuals mix in hospitals and are accompanied by family members during the night; (iii) The hospital/clinic-mixing routine and mixing with patients mode at night, where individuals mix in hospitals/clinics and are not accompanied by family members during the night.

(i) is modeled in prior studies [4, 5, 6]. These studies define coefficient of transmission ($\beta$), relative hazards of an infected individual at disease stage $X$ to symptomatic stage ($h_X$, $X$ in $\{P, A, S\}$), and relative hazards in social group $Y$ to households ($h_Y$, $Y$ in $\{H, G, C\}$). Similarly to these definitions, we define $\bar{\beta}$, $\bar{h}_X$ ($X$ in $\{P, A, S\}$), $\bar{h}_Y$ ($Y$ in $\{H, D\}$) for (ii), and $\tilde{\beta}$, $\tilde{h}_X$ ($X$ in $\{P, A, S\}$) for (iii). For (iii), $\tilde{h}_F$ is defined to be one. Based on the definition, $h_S = h_H = \bar{h}_S = \bar{h}_H = \tilde{h}_S = 1$. Note that we regard hospitalized individuals as symptomatic patients in hospitals so we will use $h_S$ ($\bar{h}_S$ or $\tilde{h}_S$) in

the calculation of their infectivities. We will explain the details of estimating these parameters in three subsections.

Furthermore, $h_{X_j}$, $\bar{h}_{X_j}$ and $\tilde{h}_{X_j}$ denote the relative hazard rate of the $j$th individual in disease stage $X$ for (i), (ii) and (iii) respectively. We have the following relationships.

$$h_{X_j} = \begin{cases} h_P & \text{if } j \text{ is a presymptomatic patient;} \\ h_A & \text{if } j \text{ is a asymptomatic patient;} \\ 1 & \text{if } j \text{ is a symptomatic/hospitalized patient;} \\ 0 & \text{otherwise;} \end{cases}$$

$$\bar{h}_{X_j} = \begin{cases} \bar{h}_P & \text{if } j \text{ is a presymptomatic patient;} \\ \bar{h}_A & \text{if } j \text{ is a asymptomatic patient;} \\ 1 & \text{if } j \text{ is a symptomatic/hospitalized patient;} \\ 0 & \text{otherwise;} \end{cases}$$

$$\tilde{h}_{X_j} = \begin{cases} \tilde{h}_P & \text{if } j \text{ is a presymptomatic patient;} \\ \tilde{h}_A & \text{if } j \text{ is a asymptomatic patient;} \\ 1 & \text{if } j \text{ is a symptomatic/hospitalized patient;} \\ 0 & \text{otherwise.} \end{cases}$$

## 3.1 Estimate of Parameters for Basic-Mixing Routine

In this subsection, we use initial $R_0$ (reproduction number), $\theta$ (proportion of transmission that occurs at presymptomatic and asymptomatic stage), $\omega$ (proportion of infections generated by individuals who are asymptomatic), $\gamma$ (proportion of transmission that occurs outside the households) and $\delta$ (proportion of transmission outside the households that occurs in the community) in Table 1 to estimate $\beta$, $h_X$ ($X$ in $\{P, A\}$) and $h_Y$ ($Y$ in $\{G, C\}$). The calibration method is used in prior studies [4, 5, 6]. We define $r_{XY}$ ($X$ in $\{P, A, S\}$ and $Y$ in $\{H, G, C\}$) as the average number of people in social group $Y$ by an individual at disease stage $X$. We can derive the following equations by definition.

$$\begin{aligned}
r_{PH} &= \textstyle\sum_{n=1}^{7} p_n(n-1)\left(1-\phi_P\left(\frac{h_P\beta}{2n}\right)\right), \\
r_{AH} &= \bar{p}_A \textstyle\sum_{n=1}^{7} p_n(n-1)\phi_P\left(\frac{h_P\beta}{2n}\right)\left(1-\phi_A\left(\frac{h_A\beta}{2n}\right)\right), \\
r_{SH} &= (1-\bar{p}_A)\textstyle\sum_{n=1}^{7} p_n(n-1)\phi_P\left(\frac{h_P\beta}{2n}\right)\left(1-\phi_S\left(\frac{\beta}{2n}\right)\right), \\
r_{PG} &= (q_1n_1+q_2n_2+q_3n_3+q_4n_4+q_5n_5)\left(1-\phi_P\left(\frac{h_Ph_G\beta}{2}\right)\right), \\
r_{AG} &= \bar{p}_A(q_1n_1+q_2n_2+q_3n_3+q_4n_4+q_5n_5)\phi_P\left(\frac{h_Ph_G\beta}{2}\right)\left(1-\phi_A\left(\frac{h_Ah_G\beta}{2}\right)\right), \\
r_{SG} &= (1-\bar{p}_A)\left((q_1n_1+q_2n_2+q_3n_3)\phi_P\left(\frac{h_Ph_G\beta}{2}\right)(1-\phi_S(0))\right) \\
&\quad +(q_4n_4+q_5n_5)\phi_P\left(\frac{h_Ph_G\beta}{2}\right)\left(1-\phi_S\left(\frac{h_G\beta}{4}\right)\right), \\
r_{PC} &= N\left(1-\phi_P\left(\frac{h_Ph_C\beta}{N}\right)\right), \\
r_{AC} &= \bar{p}_A N\phi_P\left(\frac{h_Ph_C\beta}{N}\right)\left(1-\phi_A\left(\frac{h_Ah_C\beta}{N}\right)\right), \\
r_{SC} &= (1-\bar{p}_A)N\phi_P\left(\frac{h_Ph_C\beta}{N}\right)\left(1-\phi_S\left(\frac{h_C\beta}{N}\right)\right), \\
R_0 &= r_{PH}+r_{AH}+r_{SH}+r_{PG}+r_{AG}+r_{SG}+r_{PC}+r_{AC}+r_{SC}, \\
\theta &= (r_{PH}+r_{AH}+r_{PG}+r_{AG}+r_{PC}+r_{AC})/R_0, \\
\omega &= (r_{AH}+\bar{p}_Ar_{PH}+r_{AG}+\bar{p}_Ar_{PG}+r_{AC}+\bar{p}_Ar_{PC})/R_0, \\
\gamma &= (r_{PG}+r_{AG}+r_{SG}+r_{PC}+r_{AC}+r_{SC})/R_0, \\
\delta &= (r_{PC}+r_{AC}+r_{SC})/(r_{PG}+r_{AG}+r_{SG}+r_{PC}+r_{AC}+r_{SC}),
\end{aligned}$$

where $q_i$ denotes the proportion of population in age group $i$ ($i = 1, \dots, 5$), $n_i$ is average size of peer groups for age group $i$ ($i = 1, \dots, 5$), $N$ is the total number of population, and $\bar{p}_A = 0{\cdot}25(q_1+q_2+q_3+q_5)+0{\cdot}4q_4$, which is the average probability that a presymptomatic individual does not develop symptoms. $p_n$ ($n = 1, \dots, 7$) is the probability that an individual lives in a household with $n$ members. [15] In addition, $\phi_{\rm X}(h) = E[e^{-hD_{\rm X}}]$ ($X$ in $\{P, A, S\}$) defines the probability that an infection does not occur during disease stage $X$ for a hazard of infection $h$, where the duration $D_{\rm X}$ of disease stage $X$ is defined in Table 1.

We solve the above nonlinear equations for $\beta$, $h_X$ ($X$ in $\{P, A\}$) and $h_Y$ ($Y$ in $\{G, C\}$).

## 3.2 Estimate of Parameters for Hospital/Clinic-Mixing Routine and Mixing with Family at night

Similar to previous subsection, we can solve the following nonlinear equations for $\bar{\beta}$, $\bar{h}_X$ ($X$ in $\{P, A\}$), $\bar{h}_D$.

$$
\begin{aligned}
\bar{r}_{PH} &= \textstyle\sum_{n=1}^{7} p_n(n-1)\left(1-\phi_P\left(\frac{\bar{h}_P\bar{\beta}}{2n}\right)\right), \\
\bar{r}_{AH} &= \bar{p}_A \textstyle\sum_{n=1}^{7} p_n(n-1)\phi_P\left(\frac{\bar{h}_P\bar{\beta}}{2n}\right)\left(1-\phi_A\left(\frac{\bar{h}_A\bar{\beta}}{2n}\right)\right), \\
\bar{r}_{SH} &= (1-\bar{p}_A)\textstyle\sum_{n=1}^{7} p_n(n-1)\phi_P\left(\frac{\bar{h}_P\bar{\beta}}{2n}\right)\left(1-\phi_S\left(\frac{\bar{\beta}}{2n}\right)\right), \\
\bar{r}_{PD} &= N_D\left(1-\phi_P\left(\frac{\bar{h}_P\bar{h}_D\bar{\beta}}{2N_D}\right)\right), \\
\bar{r}_{AD} &= \bar{p}_A N_D \phi_P\left(\frac{\bar{h}_P\bar{h}_D\bar{\beta}}{2N_D}\right)\left(1-\phi_A\left(\frac{\bar{h}_A\bar{h}_D\bar{\beta}}{2N_D}\right)\right), \\
\bar{r}_{SD} &= (1-\bar{p}_A)N_D\phi_P\left(\frac{\bar{h}_P\bar{h}_D\bar{\beta}}{2N_D}\right)\left(1-\phi_S\left(\frac{\bar{h}_D\bar{\beta}}{2N_D}\right)\right), \\
R_0 &= \bar{r}_{PH}+\bar{r}_{AH}+\bar{r}_{SH}+\bar{r}_{PD}+\bar{r}_{AD}+\bar{r}_{SD}, \\
\theta &= (\bar{r}_{PH}+\bar{r}_{AH}+\bar{r}_{PD}+\bar{r}_{AD})/R_0, \\
\omega &= (\bar{r}_{AH}+\bar{p}_A\bar{r}_{PH}+\bar{r}_{AD}+\bar{p}_A\bar{r}_{PD})/R_0, \\
\gamma &= (\bar{r}_{PD}+\bar{r}_{AD}+\bar{r}_{SD})/R_0,
\end{aligned}
$$

where $\bar{r}_{XY}$ ($X$ in $\{P, A, S\}$ and $Y$ in $\{H, D\}$) is defined as the average number of people in social group $Y$ by an individual at disease stage $X$ in mixing pattern (ii) and $N_D$ is the average number of patients in hospitals.

## 3.3 Estimate of Parameters for Hospital/Clinic-Mixing Routine and Mixing with patients at night

Let $\tilde{r}_{XF}$ ($X$ in $\{P, A, S\}$) be the average number of people in hospitals/clinics by an individual at disease stage $X$ in mixing pattern (iii). Similar to subsections above, we can solve the following equations for $\tilde{\beta}$, $\tilde{h}_X$ ($X$ in $\{P, A\}$), and $\tilde{h}_F$.

$$
\begin{aligned}
\tilde{r}_{PF} &= N_F\left(1-\phi_P\left(\frac{\tilde{h}_P\tilde{\beta}}{N_F}\right)\right) \\
\tilde{r}_{AF} &= \bar{p}_A N_F\phi_P\left(\frac{\tilde{h}_P\tilde{\beta}}{N_F}\right)\left(1-\phi_A\left(\frac{\tilde{h}_A\tilde{\beta}}{N_F}\right)\right) \\
\tilde{r}_{SF} &= (1-\bar{p}_A)N_F\phi_P\left(\frac{\tilde{h}_P\tilde{\beta}}{N_F}\right)\left(1-\phi_S\left(\frac{\tilde{\beta}}{N_F}\right)\right) \\
R_0 &= \tilde{r}_{PF}+\tilde{r}_{AF}+\tilde{r}_{SF} \\
\theta &= (\tilde{r}_{PF}+\tilde{r}_{AF})/R_0 \\
\omega &= (\tilde{r}_{AF}+p_A\tilde{r}_{PF})/R_0
\end{aligned}
$$

where $N_F$ is the average number of patients in hospitals/clinics.

# 4 Model Validation

We utilize the clinical attack rates for the 1957 pandemic [16] to validate our model. Clinical attack rate is the cumulative proportion of people who have ever been symptomatic. We adjust parameters as shown in Table 2. Table 3 presents the age-specific results of $R_0$ =1.5, which is in line with the estimated $R_0$ =1.5–1.7 in the 1957 pandemic [12]. The calibration procedure has been employed by several prior papers [5, 6, 9, 11, 17].

Table 2. Adjusted parameters to achieve the age-specific clinical attack rates for the 1957 pandemic.

| **Parameter** | **Original** | **Adjusted** |
|---|---|---|
| $p_A$ | 0.4 for working adults (age 19–24) and 0.25 for others | 0.35 for age 0–18, 0.47 for age 19–64, 0.65 for age 65+ |
| $S_i$ | 1.1036 for susceptible children, 0.9597 for susceptible adults, 0 if not susceptible. | 1.4236 for susceptible children, 0.8374 for susceptible adults, 0 if not susceptible. |

Table 3. Age-specific clinical attack rates for our model validation.

| **Age group** | **1957 Pandemic** [16] | **Our model with adjusted parameters** |
|---|---|---|
| Age 0–5 | 32.17% | 31.53% |
| Age 6–11 | 35.02% | 34.44% |
| Age 12–18 | 38.44% | 38.78% |
| Age 19–64 | 22.24% | 21.95% |
| Age 65+ | 10.00% | 9.86% |
| **Total** | **24.72%** | **24.40%** |

# References


1. Centers for Disease Control and Prevention. People at high risk of developing flu-related complications; 2013. http://www.cdc.gov/flu/about/disease/high_risk.htm.

2. Centers for Disease Control and Prevention. Asthma in Georgia; 2008. http://www.cdc.gov/asthma/stateprofiles/asthma_in_ga.pdf.

3. Centers for Disease Control and Prevention. Interim results: influenza A (H1N1) 2009 monovalent vaccination coverage - United States, October-December 2009; 2010. http://www.cdc.gov/mmwr/preview/mmwrhtml/mm59e0115a1.htm.

4. Wu JT, Riley S, Fraser C, Leung GM. Reducing the impact of the next influenza pandemic using household-based public health interventions. PLoS Medicine. 2006;3(9):e361.

5. Ekici A, Keskinocak P, Swann JL. Modeling influenza pandemic and planning food distribution. Manufacturing & Service Operations Management. 2013;16(1):11–27.

6. Shi P, Keskinocak P, Swann JL, Lee BY. The impact of mass gatherings and holiday traveling on the course of an influenza pandemic: a computational model. BMC Public Health. 2010;10(1):778.

7. Ferguson NM, Mallett S, Jackson H, Roberts N, Ward P. A population-dynamic model for evaluating the potential spread of drug-resistant influenza virus infections during community-based use of antivirals. Journal of Antimicrobial Chemotherapy. 2003;51(4):977–990.

8. Germann TC, Kadau K, Longini IM, Macken CA. Mitigation strategies for pandemic influenza in the United States. Proceedings of the National Academy of Sciences. 2006;103(15):5935–5940.

9. Longini IM, Nizam A, Xu S, Ungchusak K, Hanshaoworakul W, Cummings DA, et al. Containing pandemic influenza at the source. Science. 2005;309(5737):1083–1087.

10. Carrat F, Luong J, Lao H, Sallé AV, Lajaunie C, Wackernagel H. A 'small-world-like' model for comparing interventions aimed at preventing and controlling influenza pandemics. BMC Medicine. 2006;4(1):26.

11. Ferguson NM, Cummings DA, Cauchemez S, Fraser C, Riley S, Meeyai A, et al. Strategies for containing an emerging influenza pandemic in Southeast Asia. Nature. 2005;437(7056):209–214.

12. Ferguson NM, Cummings DA, Fraser C, Cajka JC, Cooley PC, Burke DS. Strategies for mitigating an influenza pandemic. Nature. 2006;442(7101):448–452.

13. Halder N, Kelso J, Milne G. Analysis of the effectiveness of interventions used during the 2009 A/H1N1 influenza pandemic. BMC Public Health. 2010;10(1):168.

14. Georgia Accrediting Commission; 2008. `http://www.coe.uga.edu/gac`.

15. United States Census Bureau Department of Commence. Census 2000; 2008. `http://www.census.gov`.

16. Chin TD, Foley JF, Doto IL, Gravelle CR, Weston J. Morbidity and mortality characteristics of Asian strain influenza. Public Health Reports. 1960;75(2):149.

17. Patel R, Longini Jr IM, Elizabeth Halloran M. Finding optimal vaccination strategies for pandemic influenza using genetic algorithms. Journal of Theoretical Biology. 2005;234(2):201–212.
