# Supplementary material for: The impact of opening dedicated clinics on disease transmission during an influenza pandemic
Source: PLoS One. 2020 Aug 6;15(8):e0236455. doi: 10.1371/journal.pone.0236455 (PMC7410326; doi:10.1371/journal.pone.0236455)
Supplement: Attachment — Submitted filename: DedicatedInfluenzaClinicsDuringaPandemic_Response.docx [file pone.0236455.s002.docx]

**Paper title:**

The impact of opening dedicated clinics on disease transmission during an influenza pandemic

**Summary:**

We thank the entire review team for their valuable feedback and comments, which have much improved our manuscript. We have addressed all the comments and revised our manuscript accordingly. Please see our point-by-point responses below and the revised manuscript for the changes.

1. Reviewer 1:
   1.1. (major comments) What are the differences between the intervention (dedicated clinics) and control (no dedicated clinics) arms? For example, are there differences in patient uptake, patient mix, or health outcomes? While the introduction mentions that dedicated clinics could help “manage people” in an influenza pandemic and can reduce the length of stay, it is unclear how specific inputs and health outcomes are different in scenarios with dedicated clinics. This lack of clarity around the central intervention makes the paper very difficult to interpret.

Thank you for your comment. The differences between intervention and control are summarized below and described in section “Methods: (ii) contact network”. A key difference is that patients with ILI visit clinics when they are open, or hospitals and associated emergency departments when they are not. Dedicated clinics also have the potential to draw worried-well; to make sure we have the worst case we assume no worried-well visits to the hospital in the control. As a result, the health outcomes were different in peak prevalence, peak day, total attack rate, hospitalization, and infections in hospitals and clinics (Tables 4-8).

To further clarify, we have added text to that section: “The difference of the two includes the parameters of patient mixing, frequency of visiting hospitals and clinics, and length of stay distributions.” to the subsection “Methods: (ii) contact network: Settings of scenarios”.

|  | Intervention:  Scenarios with some clinics | Control  Scenarios with no clinics |
| --- | --- | --- |
| Patient mix | ILI patients visited dedicated clinics; non-ILI patients visited hospitals. | Both ILI patients and non-ILI patients visit hospitals for care. |
|  | Worried-wells were present only when clinics were open. (Note, this can be interpreted as the increase in worried-wells due to open clinics.) | No worried-wells were present in hospitals. |
| Probability to visit hospitals and clinics for ILI patients v_f_; or Frequency to visit hospitals and clinics for ILI patients q_t_ | Baseline:  50% for low-risk flu patients and 100% for high-risk flu patients if they were in symptomatic stage.  For low/high frequencies, please refer to Table 2. | Baseline:  25% for low-risk flu patients and 50% for high-risk flu patients if they were in the symptomatic stage.  For low/high frequencies, please refer to Table 2. |
| Length of stay (LOS) distribution | The mean of LOS for non-hospitalized ILI patients in hospitals was 3 hours. | The mean of LOS for non-hospitalized ILI patients in hospitals was 6 hours. |

1.2. (major comments) Line 150: What does it mean to partially open a dedicated clinic? It may also be helpful to clarify what is meant by “durations” here – number of weeks the clinic is open (as opposed to daily hours of operation, etc.).

Good question. We define a partial opening of a dedicated clinic as where the clinic is open for a period of time in particular locations (specifically: which clinics to open, starting time, number of weeks to open). We have added a definition the first time the term is used.

1.3. (major comments) Positive and negative outcomes of hospitalization (either in dedicated clinics or not) should be more clearly articulated. Is there a reduction in mortality, shorter duration of infection, less interaction (and therefore transmission) to family/classmates/coworkers, but higher rates of interaction with hospital personnel and other patients? It is unclear how hospitalization impacts the pandemic in the model, and therefore whether it is needed at all.

Good point. Hospitalization is defined as admittance to the inpatient ward of the hospital, whether it is through the emergency department, a dedicated clinic, or other avenues. We specifically want to track whether mixing patients who have influenza with other patients (who may be at high-risk for severe outcomes) leads to greater hospitalizations in the second group. This is an unintended consequence that could come about in some situations. While hospitalized, patients may be isolated from their household depending (we do sensitivity analysis on this). Our focus is on the patients so we do not model healthcare personnel as a separate group but rather have some chance of transmission of patients with influenza to those who do not.

Thus in our model, hospitalized (I_H_) is one of the states in our disease progression model: the refined SEIR model, which was also used in several other studies [19-22]. The addition of this state also allows for tracking the usage of hospital beds in the simulation (for future studies).

We have added a statement to the paper to comment that hospitalizations are considered a severe outcome of flu, which is typically associated with high mortality for the flu patients and longer duration of being infectious to their contacts. The greater rate of hospitalizations in the scenarios without dedicated clinics results from the mixing patterns of ILI and non-ILI patients in hospitals.

1.4. (major comments) The interaction between infected, uninfected community members, hospital staff, and worried well is not clearly explained. When patients are discharged from the hospital, are they cured with no possibility of infecting others?

Thank you for pointing this out. A dedicated clinic is a gathering area that includes people with ILI (including those with flu plus worried-wells); during the H1N1 response some hospitals set up tents to serve these patients (and some similarities are occurring in the response to COVID-19). A hospital is a gathering area with patients admitted to the hospital (for influenza or other diseases) through the emergency department or other pathways, family members of the inpatients, and hospital staff. When dedicated clinics are not open, hospitals can also include people with ILI who are seeking care (e.g., through an emergency department) but not hospitalized. We assume that anyone who recovers from influenza during the time horizon of the model is recovered with immunity and cannot infect others. We have added text in “Methods: (i) Disease progression” to clarify this.

1.5. (major comments) Model assumptions around care and care-seeking are not clearly explained. Can an individual visit a hospital multiple times? When in the flu progression process does an individual seek care? Presumably such assumptions can influence model results.

We assume that an individual can seek care either because the individual was showing ILI symptoms (and truly does have the flu), the individual had some ILI symptoms (and was a worried-well), or for reasons unrelated to ILI (e.g., a broken leg or surgical site infection). The timing of care seeking for patients who have influenza is random within the period where they are infectious and showing symptoms.

For individuals seeking care, it is possible for them to seek care for any reason multiple times, and thus visit a hospital multiple times during the year, consistent with their profile as a low- or high-risk patient. This is to capture the fact that those who have significant comorbidities tend to visit the hospital more often than those who do not, and those same high-utilizers of care may be at greater risk of severe outcomes. See Tables 1 and 2 for more details about parameters.

1.6. (major comments) Parameter estimation: while much of these details are (and should be) in the Appendix, it would increase clarity to provide additional explanation of the data used in the main text. This would be particularly helpful in the contact network section, as it can be challenging to realistically represent age-stratified behavior by region. Pertinent details to include may be the model’s geographical resolution (zip code, county, etc.), assumptions made in assigning workplaces/schools to households, and behavioral assumptions about mixing patterns in the community (duration of contact at school/work, presence of avoidant behavior if the patient is aware of sickness, etc.) An example of a terse model and intervention description that includes these details may be found in Liu et al. “The role of vaccination coverage, individual behaviors, and the public health response in the control of measles epidemics: an agent-based simulation for California” in BMC Public Health 2015.

Thank you for that suggestion; we are happy to provide them in the body of the paper. We have added the paragraph below to the section “Methods: (ii) Contact network” of the paper.

“The model represented agents at the level of census tracts, with each assigned to a household size based on census data for the tract. During daytime, agents were assigned to schools (including daycare, preschools), workplaces, or by themselves based on their age groups (≤18, 19-64, 65+ years old, respectively). All agents interact within their household at night and in communities (e.g., grocery stores) during both day and night. If a young patient (≤18 years old) was symptomatic, the person would withdraw from schools; if an adult patient was symptomatic, the person would withdraw from work with a probability of 0.5.”

1.7. (major comments) Would it be more realistic to have the dedicated clinic open when a certain prevalence or incidence level has been reached rather than a certain number of weeks after reaching 30 initial infected cases? If the reason the initialization time was chosen that way was to account for logistical delays in setting up dedicated clinics after the critical threshold of 30 cases was reached, the authors should discuss why 30 cases is the correct value, and how logistical delays might be overcome such that the initialization day can be shortened from 10 weeks to 1 week.

The reviewer is correct that the main reason for the time delay is due to logistical challenges to setting up dedicated flu clinics, with the associated space, workers, and processes. In the past for influenza and other disease outbreaks (e.g., Ebola or Covid-19 in China), the timing of additional care delivery locations was at least partly associated with logistical leadtime. The simulation was seeded with 30 initial cases of influenza to reduce locational variation occurring from a single infected case. We have added text to the paper indicating the focus on the leadtime to set up clinics. We have also added content to the discussion about reducing the lead time to set up dedicated clinics.

1.8. (major comments) “Worried-wells were present only when dedicated influenza clinics were open” – why? When dedicated clinics are not open, do all patients who present with ILI symptoms actually have ILI? This is mentioned again later in the discussion (“Clinics have the potential for drawing the worried well but also isolate flu patients from non-ILI patients”), but it is unclear what the mechanism for this would be. Can this be clarified?

Dedicated clinics can separate influenza patients from those who may visit the hospital ED for reasons such as a broken leg or heart attack symptoms (called “non-ILI patients” as defined on page 4). Other than the associated resources, the main potential negative of a dedicated flu clinic is the possibility that it draws additional patients who would not have come to an emergency department. We assume that dedicated flu clinics are more likely to draw the worried well than if care was provided through a hospital’s emergency department. This is consistent with the experiences of several hospitals during the H1N1 pandemic in the United States. The worried well who visit a dedicated flu clinic can be thought of as the additional worried well who visit the dedicated location. These are ILI patients who do not have influenza, and we call them the “worried-well” on page 8-9.

We assume when dedicated clinics are not open, all patients denoted as having ILI symptoms in emergency departments are in the symptomatic and infectious stage of influenza (I). Of course, there can be additional patients in the emergency department with respiratory symptoms or other diseases but they are not labeled with their individual diseases.

1.9. (major comments) No mention is made of flu vaccinations. Is this included in the model, what are the assumptions around uptake, protectiveness, etc.? If not, why not?

In this paper, we assume that vaccinations are not available yet. This is consistent with a pandemic that arises unexpectedly for a novel virus. For the recent pandemic flu in 2009, the vaccine was not widely available until more than half a year after the beginning of the outbreak.

1.10. (major comments) There does not seem to be any model validation results in the main manuscript, and it would inspire more confidence in the model if there were even a basic description.

Thank you for emphasizing the importance of validation. Previously this was included in our appendix. We have now added a statement to the paper in the section Methods (i) Disease progression.

1.11. (major comments) The model is validated against 1957 pandemic data. Is it reasonable to assume that modern transmission patterns and attack rates are similar to those in 1957? We now potentially work and study much further from home (commuters), take advantage of more developed public transportation systems, potentially have higher rates of vaccination and knowledge of transmission, etc.

You are correct that there may be differences between now and 1957. In addition, we are only able to validate at an aggregate level since there is not detailed information available for individual communities. However, our process of validating against a previous pandemic is one standard approach for models of this type.

The model that is described in the paper is an adaptation of one that has been published in multiple peer-reviewed journals, studying other research questions including food distribution logistics, seasonal mutations, the role of mass gatherings on the spread of disease, and distributions of vaccines.

While our validation approach is consistent with many other researchers, we acknowledge that you are correct that there are differences for the modern world. We have added a note to the limitations section on this topic.

1.12. (major comments) Practically speaking, are the differences in outcomes across scenarios clinically or functionally important? Peak day seems to only vary by 3 days across these scenarios, and the largest difference in total attack rate is less than 5%. It is reasonable to conclude that initiation time, duration, and location do not matter? It would be helpful if these results could be translated to cases averted or total sick-days averted to understand the actual impact of these policies.

You raise a good point. We think the most important differences are in the cases averted (e.g., which can be measured by the difference in total attack rate) and in the hospitalizations for influenza (which is a measure of severe outcomes). While the difference in percentage seems small, the total number of people affected is significant, more than 100,000 for the population studied. We have revised the results to emphasize the magnitude of the numbers although we point out that a percentage representation allows one to estimate the impact for other populations. Finally, while the peak day did not vary much, we feel it is important to present the result since knowing whether it changes or not can influence resource decisions.

1.13. (major comments) If the goal of this research is to provide “guidance to stakeholders on resource allocation,” why are costs not a performance measure? The introduction also mentions the human and material resources needed for influenza clinics, but this is not considered in the model results.

You are correct that costs would be great to show; however, we found that costs varied widely according to the type of implementation of a dedicated flu clinic including both fixed cost (e.g., for the space, equipment, or overhead) and variable cost (mainly personnel potentially including providers of different types). However, we developed another way to represent the resources used, which we think could be helpful as decision makers plan ahead of or during a pandemic. For this we estimated the county-clinic days in each of the scenarios. For example, when a dedicated clinic is open for the entire horizon in all counties, this is 260*159 > 40,000 days. On the other hand, if the clinic is open for 4 weeks in 10 locations, then the number of county-clinic days is 200. We have added this data to Table 5. Thank you for leading us to this solution.

1.14. (minor) There is unconventional grammar and phrasing throughout (“thus to reduce transmission”, “Notations of constants”, present vs. past tense in introduction, etc.). A careful proofreading is recommended.

Thank you for this suggestion. We have gone over the manuscript carefully with a special eye to the verb tense and phrasing throughout. Changes are tracked in the manuscript.

1.15. (minor) Why 30 replications for each scenario?

We explored various number of replications and chose 30 based on the speed of the convergence of the performance measures and the computational time required for replications.

1.16. (minor) Should mortality be a performance measure? Is a reduction in mortality one of the benefits to hospital care for ILI patients? Does this vary with dedicated clinics?

You are correct that mortality is important. However, because it is a rare event, that is a measure that requires additional replications to show a statistical significance. We felt that hospitalizations (which represent a severe outcome associated with influenza) were a reasonable proxy instead. We have added a statement about this to the Methods section where we outline performance measures. As you point out, yes, having dedicated clinics does result in fewer hospitalizations for influenza, which is a benefit.

1.17. (minor) It would be helpful if the tables in the results sections had terse descriptions of the scenarios (instead of just “SCEN X”) to increase readability. Currently, it is very difficult to understand these tables without a lot of flipping back and forth.

Thank you for this point. We have added hierarchical descriptions to each scenario based on the key assumptions on which we focus.

1.18. (minor) It may be useful to define “worried-well” in the introduction; it is not a phrase a general audience will be familiar with. It is also unclear how long and how much contact these individuals have with ILI patients. If it is just briefly at the hospital/dedicated clinic, isn’t the transmission risk to family/community members much more significant? Why model worried-well individuals at all?

Thank you for this comment. We have added a phrase to define the term the first time it is used. Informally, transmission risk is related to the duration of mixing time along with how many people in the mixing group are infected, plus a weight associated with the transaction type. Typically household transmission is the most likely but transmission can occur in settings like workplaces and grocery stores. In the case of dedicated flu clinics, the worried-well are mixing with a population that has a high rate of infections, and it is an additional network of people not in their normal daily interactions. In some ways, it represents the worst case that a dedicated flu clinic could have, and even so we find the total infections are lower under this intervention.

1.19. (minor) Worried-well are mentioned early in the paper and appear to be an important aspect of the modeling but turn out to not the central focus of the analysis. It may guide the reader to emphasize the central question of the paper (when to open dedicated clinics) more heavily in the introduction and focus less on worried-well to avoid confusion.

Thank you for that remark. We have revised the introduction to focus more on when and how many dedicated clinics to open.

2. **Reviewer 2:**
   2.1. (major) l.42: The differences reported seem very marginal. Reading later in the paper suggests that these are the absolute difference in percentages for the various measures; however, the presentation here makes them look like (quite small!) relative differences in the measurements. Perhaps reconsider how you describe these.

Thank you for that point. We presented percentages so that it would be easy to translate the impact to other populations. However, you are correct that it does not fully communicate the impact, which can be thousands of cases averted or hundreds of hospitalizations in the population studied. We have added to the results to emphasize the impact.

2.2. (major) The model and scenarios seem to propose hospital emergency departments as the only point of presentation in the absence of dedicated clinics. Is this correct? In the UK and Australian context (with which I am more familiar) a substantial proportion of presentations are to general medical practices (GPs).

You are correct that presentations could occur to general medical practices as well as emergency departments. The CDC often recommends to call health care providers for advice if a person is mildly sick. Most of those who would present to an emergency department would have a more severe case of the illness. During the H1N1 pandemic, emergency departments were overwhelmed with cases, and for the current outbreak of COVID-19 many general practitioners are asking patients not to come in person. In addition, we consider presentations to general medical practices to be somewhat similar to community interactions, while presentations to emergency departments imply ILI patients are more likely to have interactions with high-risk patients visiting the hospital for other needs. We have added a comment to the Introduction section of the paper.

2.3. (major) l.97: "We assumed high-risk agents had a higher frequency...": high-risk and low-risk patients have not been defined yet - should do before using the terms (ie, not clear at this stage if it is an age-based charactreisation).

Thank you for pointing this out. We have revised so that we do not use the term without it having been defined.

2.4. (major) Was the probability of a high-risk agent visiting a healthcare facility greater simply because they had a higher probability of becoming infected (and hence needing to visit a healthcare facility) or was it assumed to be greater even in the absence of illness? (ie, did high-risk agents visit healthcare facilities more often even when susceptible?)

We use the term high-risk agents to refer to people who have underlying medical conditions. They visit healthcare facilities throughout the year more often than low-risk patients, and they are at higher risk for severe outcomes from the flu (e.g., because of co-morbidities).

2.5. (major) Table 1: any particular reason for using an exponential distribution for infectious durations? Given that the model is agent-based, I would think it would have been straightforward to use a more realistic distribution (along the lines of Ferguson et al 2006)

There are a number of assumptions that are used in different papers. Exponential is one that has been used by others including Ferguson et al 2005 and Wu 2006.

2.6. (major) Many details of the model seem to be omitted. For example, what is the transmission function governing whether a susceptible person becomes infected? We are told (l.107) about the different mixing groups in which transmission could presumably occur, but not how these were actually used to determine contact and transmission. What was the size of the population? The age distribution? nb: I now see that more of the model detail is provided in the Supplementary Information. Personally, I feel that some more of these details (eg about how mixing and transmission were handled by the model) could go in the manuscript itself. Or at least provide clear pointers to the reader that that material is in the SI.

Thank you for that comment. We have added a statement to the methods section that points out some of the additional information available in the appendix.

2.7. (major) l.119: What is the basis for the assumption that worried-well only exist when influenza clinics are open? Might not these individuals present to conventional GPs and hospitals in the absence of clinics?

Yes, you are correct that the worried-well may present in the absence of dedicated clinics. One can think of our approach as modeling the additional worried-well that come because the clinic is dedicated to flu, and you can also think of our approach as being the “worst-case” situation for dedicated clinics.

2.8. (major) l.167: What was the motivation for seeding with 30 initial random cases (ie rather than smaller, or clustered)? Just to minimise the impact of stochastic effects during the early stages of the outbreak?

As you highlight, seeding with 30 initial random cases minimizes the stochastic effects, which can especially occur in a particular community who happens to receive the first case. We have added a statement to the Methods section.

2.9. (major) What is the interpretation of the parameter governing opening of the clinics? Varying by week of opening suggests that it is weeks since first detection of influenza transmission (implying that this coincides with the presence of 30 cases at the beginning of the simulation. Is the timing then presumed to be a logistic constraint (ie, how long it takes to initiate the clinics - a factor over which the public health agency may have little control) or a deliberate decision made by a public health agency in order to optimise their deployment? The text seems to imply the latter, but I'm not really sure how to interpret this decision. Presumably the optimal timing of clinics being open will vary depending on the time course of the outbreak, which will vary with the transmissibility of the circulating strain (ie, R0). For example, a highly transmissible strain might benefit from earlier opening of clinics than a less transmissible strain. However, it appears that R0 was only varied for the baseline scenario and the scenario where clinics were always open, so it is not possible for the simulations run to detect this relationship. Presumably a strategy based on some endogenous epidemic indicator (eg new cases per week crossing some threshold) would be more flexible. Of course, this then touches on the importance of early estimation of R0 in order to ensure that any response is appropriate to the characteristics of the outbreak; eg,

<https://doi.org/10.1016/j.epidem.2016.01.002>

<https://doi.org/10.1371/journal.pone.0017835>

<https://doi.org/10.1016/j.epidem.2017.01.004>

<https://doi.org/10.1371/journal.pcbi.1004392>

You are correct that interventions such as dedicated clinics could be initiated either after a period of time, and/or when the epidemic reaches a particular threshold. We chose to initiate after a number of weeks to emphasize the lead time associated with setting up clinics, which we have seen as a factor in a number of response situations. The results also give some indication of how effective interventions are when used for only a period of time. As you mention, initiating after meeting a threshold on cases would work best if the early estimation of R0 was correct and when a system had a good indicator of how many true cases there were in the community. Recent results in the pandemic in the US shows that the number of true cases may not always be known. We have added a comment that another alternative could be to initiate based on a threshold.

1. [2.10. ](https://doi.org/10.1371/journal.pcbi.1004392)(major) Along the lines of my comment above, I think the results would be easier to interpret if they were reported directly in the context of the particular parameter(s) being varied, rather than the numerical scenario, which requires cross-referencing to earlier in the manuscript to interpret.

Thank you for this comment. In the revision we have changed how we reference the scenarios.

2.11. (major) Table 8 is only minimally referred to by the text. If your argument doesn't rest of the statistical significance of differences between scenarios, I'd recommend omitting this table. If these differences are important, that needs to come out in the text.

Thank you for that observation. We have added the discussion that relates to the statistical analysis.

2.12. (major) For instance, the values from Table 8 could be used to quantify the summary of findings described in the opening paragraph of the discussion (given that quantification of benefits was one of the motivations for the study).

Thank you for that point. We have added quantification of benefits in the discussion after Table 8.

2.13. (major) May also be worth mentioning the role that dedicated clinics could play in freeing up hospital resources for non-ILI related healthcare issues. From this perspective, you may wish to allow the worried-well to visit hospitals in the absence of dedicated clinics, as providing a more efficient outlet for these (WW) individuals to seek reassurance than hospitals could be another quantifiable benefit of dedicated clinics.

This is an interesting point. We have added a comment to the discussion section that highlights the advantage of freeing up hospital resources for non-influenza purposes.

2.14. (major) The proposed intervention (dedicated clinics) is modelled in isolation of other interventions (use of antivirals for treatment and prophylaxis, school closure, etc) therefore it is not possible to compare relative impact. However, could you possibly discuss how the impact of clinics might compare? For antivirals, clinics could also act as points of distribution, so there may be potential synergies.

This is an excellent point. In some of our other papers we have studied voluntary quarantine and vaccine distribution, so we have comparisons on which we can reasonably draw. We have added a comment to the Discussion section pointing out the potential impact of some of the other interventions. We have also added a comment about the potential synergies of dedicated clinics.

2.15. (minor) l.48: "visited" -> "visit"

Thanks--we have made this change.

2.16. (minor) l.96: worth including transmission diagram here for convenience

Thank you for that suggestion. We have added the diagram in the appendix.

2.17. (minor) l.142: "flu and non-ILI patients" -> "ILI and non-ILI patients" (?)

Thanks for that observation. We have changed the text.

2.18. (minor) l.169: Table 3 (and associated text) is clearly comprehensive, but actually quite difficult to parse; as it appears that all possible combinations of each parameter setting were explored for some subset of parameters, and single variations for others (the sensitivity analysis); hence it may be clearer simply to describe the purpose for varying each individual parameter.

Thank you for this suggestion. We agree that it was difficult to parse. We have chosen to change the way we denote the scenarios to make the comparisons clearer.

2.19. (minor) l.182: What was the motivation for using median/mean for the various measures?

We are examining values across a set of replications. Our statistical analysis considers both the mean and the standard deviation.

3. Reviewer 3:
   3.1. (major) It is not completely clear what the benefit is of a flu clinic or hospitalization. It seems they change transmission patterns (less mixing at home, more mixing with other patients). But, if there are more “worried well” getting care there, would it be plausible that more “real” influenza patients would get care if they know about flu clinics? (My confusion may be related to my confusion about the purpose of the pH and Vf parameters) If more patients get medical care because there are dedicated flu clinics, is there an additional benefit to those patients receiving medical care (e.g. lower mortality)? Do people who get medical care also reduce transmission (e.g. through reduced viral load from use of antivirals (or antiviral prophylaxis given to family members), or because they are educated by healthcare professionals to wear masks, wash hands, have proper cough etiquette etc?)

Dedicated flu clinics arose during the response to H1N1. The benefits include that they may attract more influenza patients than would otherwise seek care, and they may help isolate patients with an infectious disease from other patients who are seeking healthcare for other reasons. On the other hand, they require resources to staff and run, and they may attract patients who do not have influenza into the environment who may not otherwise go to the hospital.

Accounting for all of these factors, we find that on the whole it has a positive effect on outcomes rather than a negative. We also find that many of the positive outcomes can be achieved by opening for a more limited time in the highest density locations where there is greater need. There are additional benefits for which we did not account including distribution of anti-virals, education of the influenza patients, etc. We have added a comment to that effect in the Discussion. We have also tried to be clearer in our summary of the positives, negatives, and conclusions regarding dedicated flu clinics.

3.2. (minor) The tense in the first sentence (P1, line 48-49) is a little confusing: “During an influenza pandemic, many people visited health facilities to seek diagnoses and treatment [1]” Is this referring to the 2009 pandemic?

You are correct. We have changed the language in that location.

3.3. (minor) Table 2: Lc does not appear to be defined (as “levels of parameters “ for clinic switch) is that a location variable?

Thank you for the observation. We have added the definition of Lc. You are correct that it is a location variable reflecting whether there are a few clinics open or many.

3.4. (minor) Table 1 and 2: The parameters for probabilities/rates of hospitalization are confusing. From Table 1, we have pH, the probability of a symptomatic person being hospitalized (ranges between 6 and 36% based on person). But, in Table 2, we have Vf, which is the “probability to visit hospitals and clinics for flu patients” which seems to range from 25-50% in the baseline. I do not understand what the differences are here. Is Vf the (newly-increased) probability of visiting the hospital or flu clinic because of publicity about dedicated flu clinics?

Thank you for pointing this out. The pH parameter is associated with the likelihood that someone will need to be admitted as an inpatient in a hospital. We have added language in several places defining hospitalizations as inpatient admissions. The second parameter is associated with someone with influenza seeking care in either the dedicated clinic if it is open in their area, or the hospital’s Emergency Department if it is not.

3.5. (minor) Page 14: Presumably, part of the benefits of clinics are that they have shorter length of stay than hospitals. (line 134-135). Is there some evidence to suggest that clinic stays are that much shorter. Is this the Taiwan study (citation 7)?

Thanks for your question. Someone may also visit a clinic and be referred for inpatient admission to a hospital. If clinics are not open, someone may visit the emergency department of a hospital; they may or may not be admitted for an inpatient stay.

In the paper, we clarify that someone may visit a hospital (e.g., associated Emergency Department), while a *hospitalization* is an inpatient admission with a stay of at least one night.

3.6. (minor) Presumably, you compute mortality. Why not report it as a performance measure? (Are these events too rare to get statistically-significant results?)

Great question. Yes, you are correct that those events tend to be rare. We are using hospitalizations as a proxy for that. We have added a comment to that effect in the (ii) Contact network section.

3.7. (minor) As I look at the results in Table 4, it appears the odd scenarios are without clinics and the even scenarios are ones with clinics. The differences between scenarios 1, 3, 5, and 7 are differences in assumptions about the “state of nature”, not policy choices. So, to me, the impact of the policy is in the differences between the odd and even pairs (e.g. 1 vs 2, 3 vs 4, …) When I look at those, it seems to be a very small difference. So, for example, looking at 1 vs. 2, we see the total attack rate drop from 55.82% to 54.64%. It seems the other parameters (e.g. about how patients mix with other patients at night) have a dramatically larger impact on overall outcomes. That could be worth commenting on.

Thank you for this point. We have added language to the paper to indicate which ones are decisions versus nature. As you are aware, part of the reason for the other scenarios is that the state of nature is not well known, or it may vary across different hospitals.

3.8. (minor) Since Scenarios 9-29 are all variations on Scenario 2, perhaps it would be useful to add Scenario 2 to Table 5 so it is easier for readers to compare. (And, in Table 4, we compared scenario 2 to scenario 1, so it would be good to include Scenario 1 results in table 5 so we can see how they compare with no clinics. Those might be good “benchmark” comparisons. I like that you include those scenarios in Figure 4. Maybe this will be OK in the final paper when the figure is inline with the text, but reading it as a reviewer now, I am looking at the table first (before jumping to the figures in the end), and it seems like it might be nice to have those in the table too.

This is a great point. We have added Scenarios 1 and 2 to Table 5 so that it is easier to compare.

3.9. (minor) It might be interesting to comment on why you think the best week is different for the different metrics? For example, why is it that Scenario 11 has the lowest peak prevalence, but scenario 14 has the lowest total attack rate.

Interesting question. We have added some conjectures to the Discussion section about these aspects. (Please note that we reordered the scenarios to improve the flow of the paper.) In general we find that the total attack rate is lowest when the clinics are open a long time. T The peak may differ if clinics are open for a period of time then closed, which acts like a change in intervention.
