# Supplementary material for: The impact of opening dedicated clinics on disease transmission during an influenza pandemic
Source: PLoS One. 2020 Aug 6;15(8):e0236455. doi: 10.1371/journal.pone.0236455 (PMC7410326; doi:10.1371/journal.pone.0236455)
Supplement: Peer review history

# Author comment: Author response to previous submission

**DOI:** 10.1371/journal.pone.0236455.r001

4 May 2020

Attachment Submitted filename: DedicatedInfluenzaClinicsDuringaPandemic_Response.docx Click here for additional data file.

# Editor report: Decision Letter 0

**DOI:** 10.1371/journal.pone.0236455.r002

**Authors:** Yong-Hong Kuo

8 Jul 2020

The impact of opening dedicated clinics on disease transmission during an influenza pandemic

PONE-D-20-13115

Dear Dr. Swann,

We’re pleased to inform you that your manuscript has been judged scientifically suitable for publication and will be formally accepted for publication once it meets all outstanding technical requirements.

Within one week, you’ll receive an e-mail detailing the required amendments. When these have been addressed, you’ll receive a formal acceptance letter and your manuscript will be scheduled for publication.

An invoice for payment will follow shortly after the formal acceptance. To ensure an efficient process, please log into Editorial Manager at http://www.editorialmanager.com/pone/, click the 'Update My Information' link at the top of the page, and double check that your user information is up-to-date. If you have any billing related questions, please contact our Author Billing department directly at authorbilling@plos.org.

If your institution or institutions have a press office, please notify them about your upcoming paper to help maximize its impact. If they’ll be preparing press materials, please inform our press team as soon as possible -- no later than 48 hours after receiving the formal acceptance. Your manuscript will remain under strict press embargo until 2 pm Eastern Time on the date of publication. For more information, please contact onepress@plos.org.

Kind regards,

Yong-Hong Kuo

Academic Editor

PLOS ONE

Journal requirements:

1.Thank you for stating in your Funding Statement:

 [This research has been supported in part by a seed grant from Georgia Tech and by the following Georgia Tech benefactors: William W. George, Andrea Laliberte, Claudia L. and J. Paul Raines, and Richard E. “Rick” and Charlene Zalesky. This research has also been supported by Edward P. Fitts and the A. Doug Allison Distinguished Professorship.  Any opinions, findings, conclusions, or recommendations are those of the authors and do not necessarily reflect the views of the funding organizations. The funders had no role in study design, data collection and analysis, decision to publish, or preparation of the manuscript.]. 

a) Please provide an amended statement that declares *all* the funding or sources of support (whether external or internal to your organization) received during this study, as detailed online in our guide for authors at http://journals.plos.org/plosone/s/submit-now.  Please also include the statement “There was no additional external funding received for this study.” in your updated Funding Statement.

b)  Please respond by return e-mail so that we can amend your financial disclosure and competing interests on your behalf.

Additional Editor Comments (optional):

This manuscript is a revision of the original submission to PLOS ONE with reference number PONE-D-17-43889. I was the handling Editor of that submission. This manuscript was sent to the original reviewers of that submission. However, all of the three reviewers are unavailable this time. I have gone through the revision and the authors' response to the reviewers. The authors have done a serious and excellent job in this revision. I am satisfied with the revision and recommend acceptance of the paper.

Reviewers' comments:
